# Wheat Line “RYNO3936” Is Associated With Delayed Water Stress-Induced Leaf Senescence and Rapid Water-Deficit Stress Recovery

**DOI:** 10.3389/fpls.2020.01053

**Published:** 2020-07-14

**Authors:** Marlon-Schylor L. le Roux, N. Francois V. Burger, Maré Vlok, Karl J. Kunert, Christopher A. Cullis, Anna-Maria Botha

**Affiliations:** ^1^ Department of Genetics, University of Stellenbosch, Stellenbosch, South Africa; ^2^ Proteomics Unit, Central Analytical Facilities, University of Stellenbosch, Stellenbosch, South Africa; ^3^ Department of Plant and Soil Sciences, Forestry and Agricultural Biotechnology Institute (FABI), University of Pretoria, Pretoria, South Africa; ^4^ Department of Biology, Case Western Reserve University, Cleveland, OH, United States

**Keywords:** random mutagenesis, mutant wheat, drought, photosynthesis, chlorophyll fluorescence, RuBisCO, LC-MS/MS

## Abstract

Random mutagenesis was applied to produce a new wheat mutant (RYNO3926) with superior characteristics regarding tolerance to water deficit stress induced at late booting stage. The mutant also displays rapid recovery from water stress conditions. Under water stress conditions mutant plants reached maturity faster and produced more seeds than its wild type wheat progenitor. Wild-type Tugela DN plants died within 7 days after induction of water stress induced at late booting stage, while mutant plants survived by maintaining a higher relative moisture content (RMC), increased total chlorophyll, and a higher photosynthesis rate and stomatal conductance. Analysis of the proteome of mutant plants revealed that they better regulate post-translational modification (SUMOylation) and have increased expression of ribulose-1,5-bisphosphate carboxylase/oxygenase (RuBisCO) proteins. Mutant plants also expressed unique proteins associated with dehydration tolerance including abscisic stress-ripening protein, cold induced protein, cold-responsive protein, dehydrin, Group 3 late embryogenesis, and a lipoprotein (LAlv9) belonging to the family of lipocalins. Overall, our results suggest that our new mutant RYNO3936 has a potential for inclusion in future breeding programs to improve drought tolerance under dryland conditions.

## Introduction

Water deficit caused by drought conditions is a worldwide concern drastically reducing crop yield (Altieri and Nicholls, 2017). This is exacerbated by the increased vulnerability of water deficit-stressed plants to pests resulting in even greater yield loss (Botha et al., 2019). Water deficit stress was proposed as the most important abiotic factor affecting crops, because of its negative effects on both vegetative and reproductive development (Hura et al., 2010; Farooq et al., 2012; Shavrukov et al., 2017). Meteorological literature further suggests that frequency, intensity, and duration of drought periods will increase in the coming years (Dai, 2011; Schubert et al., 2016; Spinoni et al., 2017; Botha et al., 2019, and references within) and new biotechnological advances are one of the tools required to increase crop productivity (Botha et al., 2019 and references within; Wulff and Dhugga, 2019).

Mutational breeding is a long-known and well-tested technology that increases genetic diversity in a relatively short time span (Muller, 1927; Pacher and Puchta, 2017). Mutagenesis can be induced through exposure to chemicals (e.g., sodium azide, ethylmethanesulfonate [EMS], etc.) or irradiation with X-rays and gamma rays. Effective mutational breeding to select plants for drought tolerance is difficult, as it is a polygenic controlled trait that is heavily regulated by genotype and environment interactions (Vassileva et al., 2011; Simova-Stoilova et al., 2016). In recent years, over 1,500 commercially available cereal varieties are the product of mutagenesis, with most varieties available from rice (828), barley (312) and wheat (282) (FAO/IAEA Mutant Varieties Database, 2018; https://www.iaea.org/resources/databases/mutant-varieties-database). A good example of such a wheat mutant is *tasg1*, developed through EMS, and expressing a “stay-green” phenotype (Tian et al., 2012). The *tasg1* mutant exhibited a distinct delayed senescence under both normal and drought stress conditions, as indicated by slower degradation of chlorophyll and decrease in net photosynthetic rate when compared to its wild type (WT) progenitor. The *tasg1* mutants maintained more integrated chloroplasts and thylakoid ultrastructure than did WT plants under drought stress. The authors suggested that a lower malondialdehyde content and higher antioxidative enzyme activities (ascorbate peroxidase, catalase, peroxidase) in *tasg1* was the casual factor that allowed the plants to perform better under drought stress. However, despite these suggestions, supporting evidence was limited as the observations were mostly based on measuring chlorophyll fluorescence, selected enzymatic activities, and chlorophyll structure using microscopy.

Plants respond to water deficit stress following any combination of four strategies, namely drought avoidance (Franks, 2011); drought tolerance; drought escape and drought recovery. Of particular interest is drought recovery, which defines the plant's ability to recover from dehydration and loss of turgor pressure as a result of the induced water deficit stress, thereby resuming growth and eventually producing seed (Luo, 2010; Fang and Xiong, 2014).

Photosynthetic activity in plants has been shown to be a trait that is highly responsive to water deficit stress (Singh et al., 2014; Serba and Yadav, 2016; Perdomo et al., 2017). In wheat, a direct correlation exists between imposed water-deficit stress and decreases in photosynthetic rate, leading to changes in intercellular CO_2_ concentration, stomatal conductance, and transpiration rate (Subrahmanyam et al., 2006; Balla et al., 2014; Sharifi and Mohammadkhani, 2016; Perdomo et al., 2017; Senapati et al., 2018). Water deficit stress negatively affects maximal quantum yield of PSII photochemistry (F_v_/F_m_) (Tian et al., 2017), and damages the oxygen-evolving complex of PSII and its reaction centers (Aro, 2004; Murata et al., 2007; Tian et al., 2017). Damage to PSII centers are often due to impairment in ATP synthesis as a consequence of a decline in electron transport rate, which leads to a reduction in ATP availability and thus to a concomitant reduction in ribulose-1,5-bisphosphate (RuBP) regeneration (Lawlor and Cornic, 2002; Ma et al., 2006; Perdomo et al., 2017).

Ribulose-1,5-bisphosphate carboxylase/oxygenase (RuBisCO) is the main protein involved in CO_2_ assimilation. Reports vary from no changes in the protein (Panković et al., 1999; Pelloux et al., 2001), to significant declines in the enzyme due to water deficit caused by drought conditions (Zhou et al., 2007; Galmés et al., 2011). Collectively, data suggest that RuBisCO levels and activity are influenced by the extent of water deficit stress and appears to be highly species-specific (Tezara, 2002; Bota et al., 2004). Water deficit stress decreased the amount of RuBisCO in maize and rice, but not in wheat (Perdomo et al., 2017). This decline in RuBisCO content subsequently led to a decline in carbon assimilation and an imbalance between photosynthesis and electron availability, with the resultant accumulation of reactive oxygen species (ROS), primarily hydrogen peroxide (Reddy et al., 2004).

Accumulation of ROS due to disturbance of cellular homeostasis can be countered through a unique set of biochemical mechanisms that detoxify ROS (Abid et al., 2016; Foyer et al., 2017; Foyer, 2018; Foyer et al., 2018; Noctor et al., 2018). High concentration of ROS is deemed extremely toxic to cells leading to oxidative bursts and potential cell death (Demidchik, 2015). Proteins involved in oxidative stress are peroxidases (POX), Glutathione S-transferases (GST), ascorbate peroxidases (APX), thylakoidal ascorbate peroxidase (tAPX), copper‐zinc superoxide dismutases (SOD), catalases (CAT), and glutathione peroxidases (GPX). These enzymes are often referred to as ROS-scavenging enzymes (Sairam et al., 2005; Asensi-Fabado and Munné-Bosch, 2010). Increase in oxidative signals from photosynthesis and associated redox-sensitive proteome, provide cells with capacity to monitor photosynthetic electron flow and counteract over-reduction or over-oxidation. It further also produces redox regulatory networks that facilitate sensing and response to changes in environmental conditions (Martin and Sies, 2017; Foyer, 2018).

Water deficit stress alters the plants' metabolome (i.e., due to the production of newly synthesized metabolites), which may modify cellular structure, change the plant's metabolism and phenotype (Kosová et al., 2015). Michaletti et al. (2018) demonstrated the fluctuation of major metabolites in two wheat varieties that differ in their sensitivity to water deficit stress. They found that amino acid metabolism correlates to water deficit stress sensitivity after studying nine metabolic pathways. Specifically, N-containing compounds (e.g. betaine, glycine, and proline), and sugars (e.g. sucrose, trehalose, fructans) often accumulate in the cytoplasm as osmoprotectants contributing to water deficit stress tolerance.

Proteomics is a powerful tool to understand plant reactions to drought stimuli especially in a hexaploid crop such as wheat. Comparative proteomics analysis is deemed as an effective strategy to identify crucial proteins, however in crops such as wheat, it becomes very difficult because of the large genome size. Techniques such as Liquid Chromatography with tandem mass spectrometry (LC-MS/MS) provide unique indications of expressed proteins at a given time point. However, it does not give an indication of the activity of a given protein, and thus, technologies such as LC MS/MS should be combined with activity assays to provide a more holistic demonstration of important proteins and their activities in context of water stress tolerance. Water deficit is known to induce changes in the proteome of plants because of protein breakdown catalyzed by proteases (Vierstra, 1993; Hieng et al., 2004; Simova-Stoilova et al., 2010). Proteases are ubiquitously required for readjustment of a plant's metabolic status, through the method of protein turnover, to remobilize nutrients to counter environmental shifts and maintain developmental processes (Nelson and Millar, 2015). Known protein turnover processes involved in this readjustment include ubiquitination, phosphorylation, and SUMOylation with the latter referring to the post-translational modification of protein substrates through the covalent conjugation with the SUMO (Small Ubiquitin-like Modifier) peptide. This modification is reminiscent of ubiquitination, even though it has its own set of homologous enzymes (Ranieri et al., 2018). The conjugation of SUMO on a protein is reversible (de-SUMOylation) where SUMO proteases (clan cysteine proteases) cleaves SUMO conjugates of the targeted proteins (Guerra et al., 2015; Benlloch and Lois, 2018; Morrell and Sadanandom, 2019 and references within).

Even though water deficit induces major changes in the biochemical processes of plants, drought is often an episodic event. Once soil moisture is restored (i.e., by irrigation or rain fall), plants regain normal physiological functionality (i.e., water transport and turgor pressure, stomatal conductance, photosynthetic activity, etc.) and grow to maturity to produce seed. For plants experiencing prolonged periods of water deficit, this does not happen naturally, and only selected plants display a “recovery” phenotype whereby this can be reversed after severe water deficit (Abid et al., 2018). In order to better understand the underlying genetic mechanisms enabling plants to delay senescence and recover after being completely dehydrated, we compared physiological responses, metabolic and enzymatic activity, and changes in the proteome of a water deficit stress-sensitive wheat line (Tugela DN) with its near-isogenic mutant line (RYNO3936) that express a combination of water deficit stress avoidance and recovery phenotypes. An improved understanding of such a phenotype will ease introducing these traits into breeding programs for enhanced drought-tolerant phenotypes and its future discovery in mutant breeding programs.

## Materials and Methods

### Water-Deficit Treatment, Plant Phenotyping, Plant Biomass, and Soil Relative Moisture Content

Random mutagenesis was performed as previously described (Mbwanjii, 2014; Botha et al., 2017). In brief, mutant RYNO3936 was developed using chemically induced mutagenesis by exposing seed of a red hard winter wheat cultivar, Tugela DN to 1 mM Sodium azide for 2 h, where after the treated seed was planted in trays containing equal amounts of substrate (sand: soil) and grown in a greenhouse at temperatures between 20°C and 26°C. After a month of growth, water was withheld, and plants selected for water deficit tolerance under low nitrogen regimes. The resultant mutant, RYNO3936 was then selfed for six generations to retain the water deficit stress tolerant trait (Mbwanjii, 2014; Botha et al., 2017).

Seeds of the WT parent (Tugela DN) and mutant RYNO3936 were grown in a greenhouse with natural day/night temperature at 23 ± 3°C (Welgevallen Experimental Farm, Stellenbosch University, South Africa). Seeds were planted in pots [dimensions: 25 cm (diameters) × 30 cm (height)] filled with equal amounts of sand and crusher dust (1:1). A total of 30 pots (15 per control and 15 per mutant) arranged in a randomized complete block design was used, with each pot containing 5 seeds. The plants were watered daily using a fully automated system containing nutrients (Multifeed™, South Africa). All pots were regularly assessed to ensure that a constant gravimetric reading of 80% was maintained, until plants reached the final extension stage (58–65 days after germination) corresponding to phase 45 of Zadoks' scale (Zadoks et al., 1974; Vendruscolo et al., 2007). From this stage onwards, the watering was withheld, and water deficit stress induced. Day 0 measurement was recorded under irrigation (gravimetric reading of 80%). Water was then withheld for 14 consecutive days or until soil moisture reached 21% to 24%, with measurements collected on days 7 and 14. The plants were then subjected to rehydration for 7 days during which a gravimetric reading of 70% was maintained, when the last set of data was collected (i.e., day 21 denoted “re-watered”).

Plant growth analysis (i.e., plant height, and flag leaf length and width) were assessed as previously described (Aase, 1977) using a line gauge (unit of measurement in mm). For plant height, all the individual tillers of the plant were measured from the ground to the tip of the tallest tiller of the plant (n = 20). The relative moisture content (RMC) was calculated according to the following formula: RMC (%) = (fresh weight − dry weight)/(turgid weight − dry weight) × 100 (Sade et al., 2014; Sade et al., 2015). The flag leaf was removed and kept in distilled water for ± 4 h to achieve full turgidity. The leaf dry weight was measured after keeping the turgid leaf at 80°C in an oven for 16 h. The RWC was tested at days 0, 7, and 14 after induction of water deficit stress and then 7 days after re-watering (day 21). The RWC was measured using three similar-sized leaves and six replicates for each treatment. Soil samples (n = 3) to 150 mm depth were also collected, and the wet soil mass was determined through drying the soil in an oven at 105°C for 48 h after which the soil was weighed and the gravimetric soil moisture content determined (Black and Power, 1965).

### Stomatal Conductance, Chlorophyll Fluorescence, and Chlorophyll Content Measurement

Chlorophyll fluorescence and stomatal conductance were measured at respective time points (days 0, 7, 14, and after re-watering, day 21) as previously described (Le Roux et al., 2019). Stomatal conductance (gsw) was measured at three positions on each leaf using three independent plants (n = 9) with a porometer (model SC-1, Decagon Devices Inc., Pullman, WA, USA) following the manufacturer's instructions. Rate of photosynthesis was measured according to Strasser et al. (2004) making use of chlorophyll fluorescence induction transients (O-J-I-P), using a hand-held Chlorophyll Fluorometer (model: OS-30P; Manufacturer: Opti-Sciences, Inc., United States). Dark adaptation clips were applied to leaves for 20 min (prior to reading) to achieve a flush out of assimilates. Technical repeats for both instruments were recorded at different places from the tip to the base of the flag leaf to represent the entire leaf surface. All measurements were taken at the onset of the water deficit stress treatment (day 0), then at days 7 and 14 after induction of water deficit stress. At day 14, plants were re-watered and then watered on a daily basis to ensure full recovery. Measurements were then taken on day 21 (denoted “re-watered”). Chlorophyll concentrations were quantified and calculated according to Arnon (1949) using the SmartSpec™ Plus BioRad.

### SDS-PAGE Electrophoresis and Western Blot Analysis

Total protein was extracted and separated using the Mini-Protein TGX gradient gel (4%–15%) as previously described (Le Roux et al., 2019). The Bio-Rad protein assay reagents with bovine albumin as the standard (Bio-Rad Laboratories Inc., Hercules, CA) was used for determination of protein concentration (Bradford, 1976), and quantified using a Glomax Spectrophotometer (Promega, Sunnyvale, CA) (Rylutt and Parish, 1982).

Western blot analyses were conducted using a Bio-Rad Trans-Blot^®^ SD semi-dry transfer cell apparatus and polyvinylidene difluoride membranes (Hybond-P, Amersham Biosciences). The membranes were blocked with 3% bovine serum albumin (BSA) and probed with polyclonal large (RbcL) and small (RbcS) RuBisCO Subunits (RbcL and RbcS, 1:50000; Botha and Small, 1987) and human anti-SUMO1 monoclonal antibody (1:2500) (UBPBio, Aurora, USA) diluted in buffered saline containing 3% BSA. Detection employed alkaline phosphatase conjugated Donkey Anti-Mouse (Abcam) (1:2500) or goat anti-rabbit (1:7000) (Sigma-Aldrich, St. Louis, MO, USA) antibodies in conjunction with nitro blue tetrazolium and 5-Bromo-4-chloro-3-indolyl phosphate (Sigma-Aldrich, St. Louis, MO, USA).

### Protease Determination

Leaf tissue was ground after being flash frozen in liquid nitrogen; the powder was added to cold 0.1 M citrate-phosphate buffer (pH 5.6) containing 10 mM L-cysteine. A centrifugation step was included at 25,000 × *g* for 20 min at 4°C. The supernatant was electrophoresed using a gradient acrylamide gel (5–15%). The gradient gel was prepared using the Hoefer™ SG Series Gradient Makers system as described by Le Roux et al. (2019). A gel equilibration step was conducted by pre-electrophoresis for 60 min at 50 V in the gel buffer storage condition at 4°C. Samples (80 mg) were loaded with and without addition of the cysteine proteinase inhibitor E64 (Barrett et al., 1982; Matsumoto et al., 1999). Proteins were separated at 15 mA for 2 h. After electrophoresis, the gels were meticulously removed from the glass plates and washed three times in a renatured buffer (5 mM cysteine and 2.5% v/v Triton-X 100) and subsequently incubated in developing buffer (0.5% v/v Triton-X 100, 50 mM Tris–HCl, pH 7.5 and 5 mM CaCl_2_, 1 mM ZnCl_2_, 10 mM cysteine) for 24 h. The gels were stained with Coomassie R-250 and de-stained until clear zones were visible against the dark blue background (Le Roux et al., 2019).

### Amino Acid Extraction and Quantification

Amino acid extraction and quantification were conducted as previously described (Le Roux et al., 2019). In brief, leaf material was dried out in an oven at 60°C for 24 h, where after samples were ground to a powder and 0.5 ml of 6 M HCl containing norleucine (250 ppm) added as an internal standard. AccQ.Tag derivatives of extracted amino acids were generated using the AccQ.Tag Ultra Derivatization Kit following the manufacturer's instruction (Waters). Derivatized amino acids were analyzed using an Acquity UPLC system equipped with a binary solvent delivery system and an auto sampler. For separation an AccQ.Tag Ultra column (100 9 2.1 mm) (Waters) was used. Derivatized amino acids were detected at 260 nm using a photo diode array detector. Amino acids in the samples were identified by co-elution with amino acid standard H (Pierce) and commercially available individual amino acids (Sigma). Concentration of amino acids in each sample was calculated based on the peak areas and calibration curves generated with commercial standards.

### Proteome Analysis

#### Protein Extraction, Quantification, and Digestion

Leaf protein was extracted using a modified method to that which was previously described (Damerval et al., 1986; Wang et al., 2006; Wang et al., 2007). In brief, after treatment the leaf tissue was ground into fine powder in liquid nitrogen and transferred to a falcon tube. To this, 20 ml of cold (−20°C) extraction buffer (10% w/v TCA/acetone containing 0.07% β-mercaptoethanol (β-ME) was added and homogenized by vortexing vigorously for 20 s. Where after the tube was incubated at −80°C overnight to allow complete precipitation of proteins. This procedure was repeated for each of the treatments. After the overnight incubation, the tubes were centrifuged at 5,200*g* for 30 min at 4°C and the supernatant removed. Three acetone washes were consecutively performed by adding 5 ml of ice-cold acetone (−20°C) containing 0.07% β-ME, vortexing the tube briefly, centrifuging at 5,200*g* for 15 min, and again removing the supernatant. After completing the third wash, the supernatant was removed, and the tube was centrifuged for another 10 min, where after the remaining supernatant was removed using a pipette. The pellet was lyophilized for 2 h. The lyophilized samples were stored at −80°C till further use.

### iTRAQ Labeling and SCX Fractionation

#### Protein Digestion

The iTRAQ-labeling and analyses were conducted as previously described (Wang et al., 2016). In brief, an aliquot containing 100 μg of solubilized protein in 100 mM triethylamonium bicarbonate (TEAB, Sigma) containing 4M Guanidine-HCl, was reduced with tris-carboxyethyl phosphine (TCEP, Sigma) at 60°C prior to cysteine residues being thiomethylated with methane methylthiosulfonate (MMTS, Sigma). The reduced and thiomethylated sample was digested with Trypsin Gold (Promega, Madison, USA) (protein/trypsin = 20:1) after a 10 times dilution at 37°C for 18 h. The peptides dried under vacuum and resuspended for desalting in 2% acetonitrile/water containing 0.1% formic acid. Residual digest reagents were removed using an in-house manufactured C_18_ stage tip (Empore Octadecyl C_18_ extraction discs; Supelco). The 20 µL sample was loaded onto the stage tip after activating the C_18_ membrane with 30 µL methanol (Sigma) and equilibration with 30 µL 2% acetonitrile/water; 0.05% TFA. The bound sample was washed with 30 µL 2% acetonitrile/water; 0.1% FA before elution with 30 µL 50% acetonitrile/water 0.1% FA. The eluate was evaporated to dryness. The dried peptides were dissolved in 20 µL 2% acetonitrile/water; 0.1% FA for LC-MS analysis

### LC–ESI-MS/MS Analysis Based on the Thermo Scientific Fusion Tribrid System

Liquid chromatography was performed on a Thermo Scientific Ultimate 3000 RSLC equipped with a 0.5 cm × 300 µm C_18_ trap column and a 35 cm × 75 µm in-house manufactured C_18_ column (Luna C_18_, 3.6 µm; Phenomenex) analytical column. The solvent system employed was loading: 2% acetonitrile/water; 0.1% FA; Solvent A: 2% acetonitrile/water; 0.1% FA and Solvent B: 100% acetonitrile/water. The samples were loaded onto the trap column using loading solvent at a flow rate of 15 µL/min from a temperature controlled autosampler set at 7°C. Loading was performed for 5 min before the sample was eluted onto the analytical column. Flow rate was set to 500 nl/min and the gradient generated as follows: 2.0% to 10% B over 5 min; 5% to 25% B from 5 to 50 min using Chromeleon non-linear gradient 6, 25% to 45% from 50 to 65 min, using Chromeleon non-linear gradient 6. Chromatography was performed at 50°C and the outflow delivered to the mass spectrometer through a stainless-steel nano-bore emitter.

The Thermo Scientific Fusion mass spectrometer was equipped with a Nanospray Flex ionization source. The sample was introduced through a stainless-steel emitter. Data was collected in positive mode with spray voltage set to 2 kV and ion transfer capillary set to 275°C. Spectra were internally calibrated using polysiloxane ions at m/z = 445.12003 and 371.10024. MS1 scans were performed using the orbitrap detector set at 120 000 resolution over the scan range 350 to 1650 with AGC target at 3 E5 and maximum injection time of 40 ms. Data was acquired in profile mode.

MS2 acquisitions were performed using monoisotopic precursor selection for ion with charges +2-+6 with error tolerance set to ± 0.02 ppm. Precursor ions were excluded from fragmentation once for a period of 30 s. Precursor ions were selected for fragmentation in HCD mode using the quadrupole mass analyzer with HCD energy set to 32.5%. Fragment ions were detected in the orbitrap mass analyzer set to 15 000 resolution. The AGC target was set to 1E4 and the maximum injection time to 45 ms. The data were acquired in centroid mode.

The raw files generated by the mass spectrometer were imported into Proteome Discoverer v1.4 (Thermo Scientific) and processed using the SequestHT algorithm included in Proteome Discoverer. Data analysis was structured to allow for methylthio as fixed modification as well as NQ deamidation (NQ), oxidation (M). Precursor tolerance was set to 10 ppm and fragment ion tolerance to 0.02 Da. The database used was the murine taxonomy database obtained from Uniprot with the sequence of amyloid beta A4 P05067 added. The results files were imported into Scaffold v1.4.4 and identified peptides validated using the X!Tandem search algorithm included in Scaffold. Peptide and protein validation were performed using the Peptide and Protein Prophet algorithms. Protein quantitation was performed by first performing a t-test on the paired data and applying the Hochberg-Benjamini correction (Benjamini and Hochberg, 1995).

### Bioinformatics Analysis

When comparing differential expression of peptides between treatments (i.e., days 0, 7, 14, and 21), data obtained were analyzed using Scaffold Viewer 4 proteomics software (http://www.proteomesoftware.com/products/scaffold/; Searle, 2010) by comparing all treatments with each other. The Benjamini-Hochberg multiple testing adjustment was applied in order to control the comparison-wide false discovery rate (Benjamini and Hochberg, 1995). Sequences representing the peptide were subjected to Blast2GO (Conesa et al., 2005) analysis to obtain the representing genes, as well as gene ontologies and functional categories. A *P*-value ≤ 0.05 was used as the threshold to determine the significant enrichments of GO and KEGG pathways.

### Clustering and Data Analysis

Resulting peptide intensity signals were first normalized using the Cluster program (Eisen et al., 1998), with mean-centering applying Spearman's rank correlation. A cluster image representing groups of differentially expressed peptides that share similar expression patterns was generated from the normalized data and visualized with Java TreeView (Saldanha, 2004).

### Enzyme Measurements and Protein Determination

Extraction of enzymes was performed as described in Botha et al. (2014). All enzyme activity measurements were conducted using three biological repeats (n = 3) and conducted in triplicate (n = 9). Peroxidase activity was determined following a modified method of Zieslin and Ben-Zaken (1993) and expressed as mmol tetraguaiacol min^−1^ mg^−1^ protein. Glutathione S-transferase (GST) enzyme activity was measured as described by Venisse et al. (2001), with the formation of GS-DNB conjugate measured at 340 nm. GST activity was expressed as mmol GSH min^−1^ mg^−1^ protein.

### Statistical Analyses

All data were collected using three biological repeats (n = 3) with measurements done in triplicate (n = 9). Mean values are presented with their standard deviation (SD) and analyzed using Graphpad Prism software version 5.0 (http://www.graphpad.com/scientific-software/prism/) (Motulsky, 2007). Statistical validation and significance (*P* ≤ 0.05) were determined with analysis of variance (ANOVA) followed by post-t Dunnett's, or Turkey or Bonferonni tests (Amstrong, 2014).

## Results

### Plant Phenotypes, Physiological Response, and Reproduction Under Water Deficit

**Figure 1:**
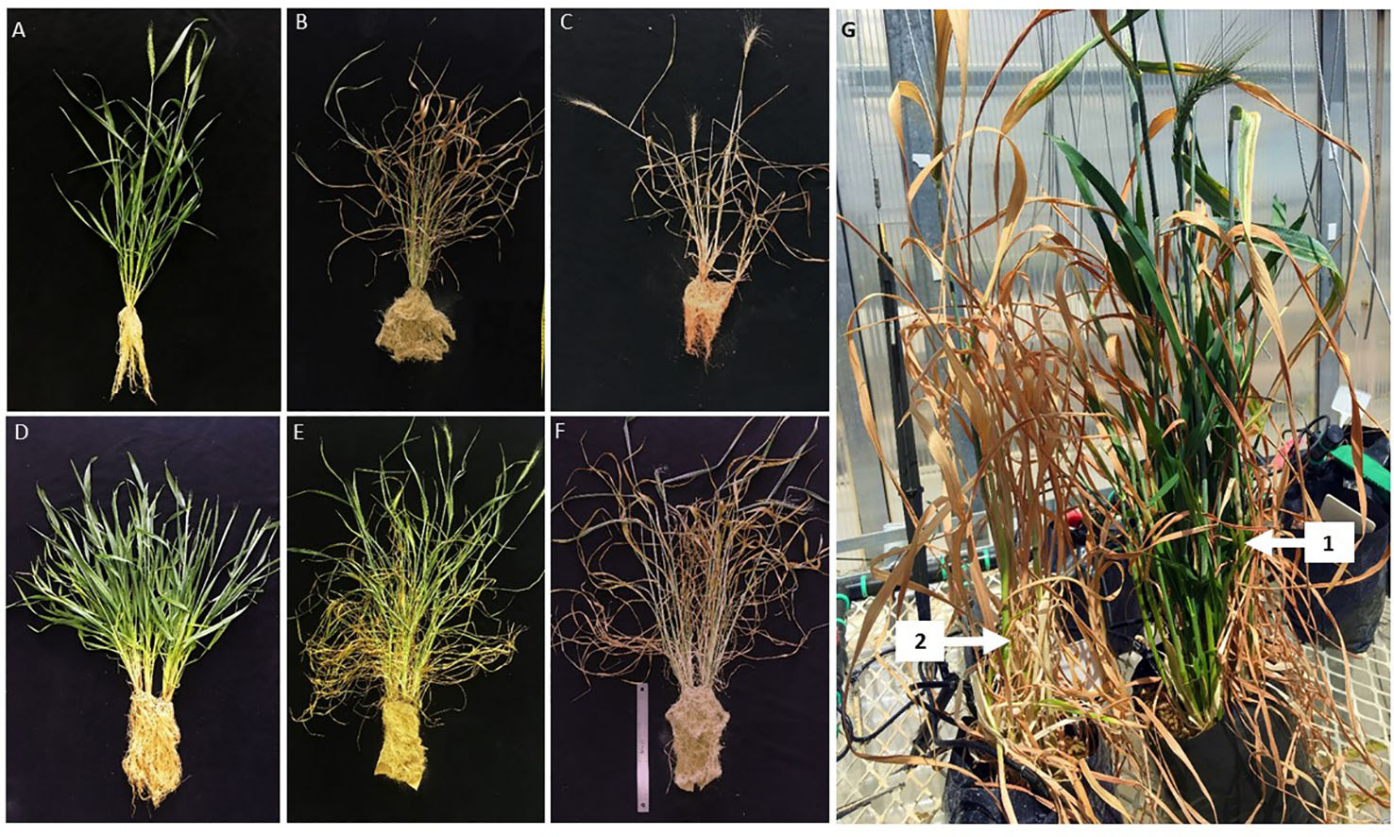
Phenotypic response of WT Tugela DN and Mutant RYNO3936 wheat prior to **(A, D)** and after induction of water stress **(B, C**; **E, F)**. Where **(A–C)** is WT Tugela DN wheat at day 0 **(A)**; day 7 **(B)**; and day 14 **(C)**. While **(D, E)** is Mutant RYNO3936 wheat at day 0 **(D)**; day 7 **(E)**; day 14 **(F)** and re-watering **(G)** the arrow shows initiation of recovery 2 and fully recovery 1.

**Figure 2:**
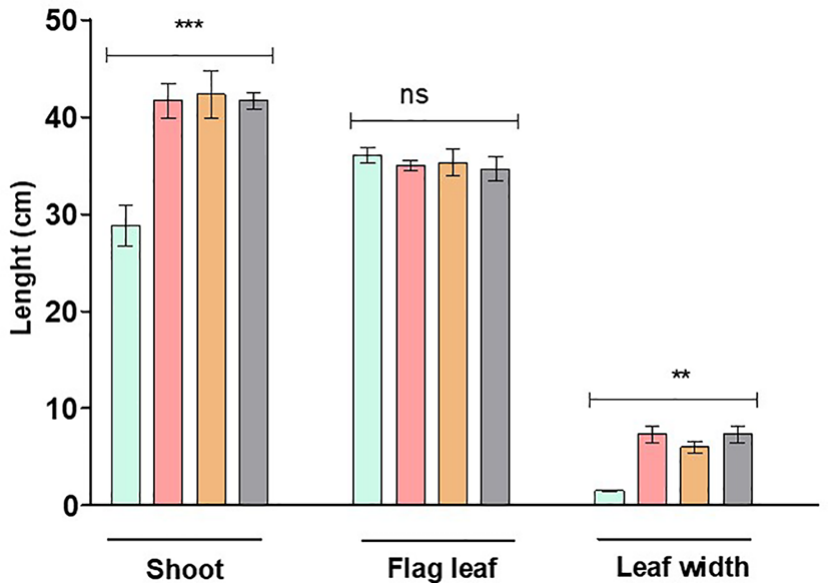
Plant height as well as flag leaf length and width of WT Tugela DN and Mutant RYNO3936 prior to exposure to water stress. Capped bars above means represent ± SD of three replicates. Asterisks above columns means denote the significant differences compared with WT Tugela DN for a single mutant line. **P ≤ 0.01; ***P ≤ 0.05. ns, nonsignificant.

The mutant wheat line RYNO3936 visually displayed a “bushy” phenotype with longer shoots and broader leaves, and more tillers and roots when compared to its WT parent (**Figures 1A, D** and **2**). After induction of water deficit, the WT Tugela DN was visually wilted after 2 days of water deficit stress, and dead after day 7 (**Figures 1B, C**). In contrast, the mutant wheat line RYNO3936 visually displayed signs of stress only after day 7 of induced water deficit stress and lasted much longer than the WT Tugela DN plants (**Figures 1E, F**). Re-watering of the mutant wheat line RYNO3936 after day 14, resulted in partial to full recovery of the mutant plants, but not the control (day 21) (**Figure 1G**).

When growth and reproduction of the mutant wheat line RYNO3936 was compared with its WT Tugela DN parent, it took significantly longer (148.0 ± 4.0 *vs*. 100.5 ± 9.5 days) to reach the heading phase under well-watered growth conditions, but headed much sooner (92.0 ± 11.0 days) under water deficit conditions (**Table 1**). Under well-watered growth conditions, mutant wheat line RYNO3936 produced significantly more heads (7.0 ± 2.0) and seeds per plant (120.0 ± 33.0) when compared with its WT parent (heads = 2.0 ± 1.0; seed = 32 ± 10.0). With the induction of water deficit stress, the WT parent died after 7 days and failed to reproduce. Under water deficit stress, the mutant wheat line RYNO3936 yielded equivalent to what the WT parent yielded under well-watered growth conditions (**Table 1**).

**Table 1:** The response of WT Tugela DN and Mutant RYNO3936 to water deficit stress.

Genotype	Treatment (days)	Days to heading (days)	Anthesis from heading date (days)	Heads per plant (number)	Seeds per plant (number)
**RYNO3936**	Control	148.0 ± 4.0	16.0 ± 4.0	7.0 ± 2.0	120.0 ± 33.0
	Water stressed	92.0 ± 11.0	7.0 ± 2.0	2.5 ± 1.5	34.0 ± 6.0
**WT**	Control	100.5 ± 9.5	12.5 ± 1.5	2.0 ± 1.0	32.0 ± 10.0
	Water stressed	nd	nd	nd	nd

Indicated are days to heading, days to anthesis, number of heads and seeds produced prior (0 day) and after induction of water deficit stress (days 7 and 14) (± = standard deviation) (n = 3). nd, WT Tugela DN dies prematurely and produced no seed.

**Figure 3:**
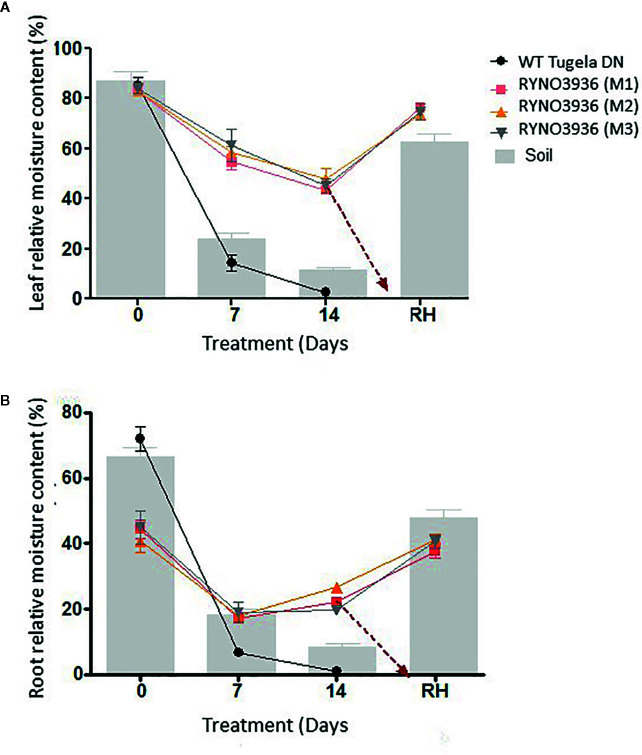
Comparative analysis of relative moisture content (RMC) measured in the leaves **(A)** and roots **(B)** prior to and after induction of water stress. The gravimetric readings of the soil are superimposed against the RMC. Indicated are the RMC of WT Tugela DN and Mutant RYNO3936 emphasizing how they maintain RMC under deficient soil moisture. Error bars indicate SD (n = 9) and significance was set at p ≤ 0.05.

Even though the relative moisture content (RMC) in the leaves was similar between the mutant and WT plants before the induction of water deficit stress, RMC declined significantly (*P* < 0.05) in WT Tugela DN, but not in the mutants, with the induction of water deficit stress (**Figure 3A**). This decrease in RMC coincided with declines with soil moisture content (**Figure 3A**). At Day 7, the mutant lost nearly 21% of its RMC, whereas the WT lost 65% with both having access to a soil moisture content of ± 24%. After re-watering at day 14, the mutant RYNO3936 took three days to display greening of leaves, with some leaves returning to its natural green color after approximately a week after regaining most of its RMC ( ± 76%). However, not all leaves could recover in the mutant plants from water stress-induced damage as can be observed in **Figure 1G**.

RMC of the WT Tugela DN plant roots was significantly higher ( ± 70%) than that of the mutant (± 45%) pre-induction of water deficit stress (day 0) but declined to below 10% after induction of water stress (**Figure 3B**). In contrast, even though the RMC in the roots of mutant RYNO3936 decreased about ±50%, it remained at the ±20% level throughout the water deficit experiment and regained RMC with re-watering to return to pre-water deficit values.

**Figure 4:**
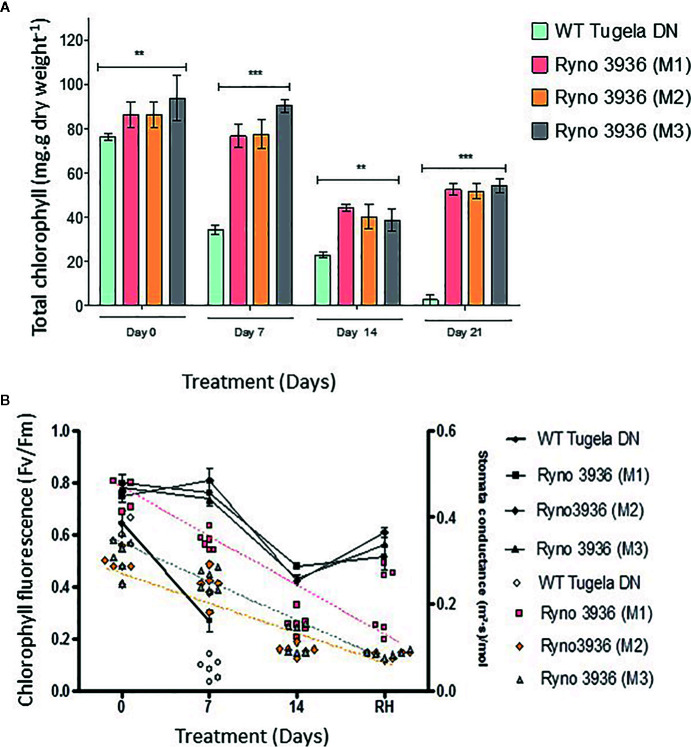
**(A)** Total chlorophyll measured in the WT Tugela DN and Mutant RYNO3936. prior to (day 0) and after exposure to water stress (days 7 and 14). Capped bars above means represent ± SD of three replicates. Asterisks above columns means denote the significant differences compared with WT Tugela DN for a single mutant line. **P ≤ 0.01; ***P ≤ 0.05. **(B)** Rate of photosynthesis (F_v_/F_m_) (line graph) and stomatal conductivity (scatter plot) prior to (day 0) and after exposure to water stress (days 7 and 14). Photosynthesis significance was determined by p ≤ 0.005 where n = 6 and error bar indicate SD.

Chlorophyll content decreased in both plants with the WT Tugela DN showing a greater loss in chlorophyll (± 50%) within the 7 days after induction of water deficit, which is much sooner than in the mutant RYNO3936 plants (**Figure 4A**). Chlorophyll content increased with re-watering, while the WT plant suffered a complete loss of chlorophyll with no recovery despite re-watering. A significant (p < 0.05) decline in chlorophyll fluorescence, as a measure of PSII efficiency (Schreiber et al., 2003; Strasser et al., 2004), was measured in both the WT Tugela DN and mutant RYNO3936 after induction of water deficit stress (**Figure 4B**). This decrease in chlorophyll fluorescence was less in the mutant wheat line than in its WT parent and recovered in the mutant RYNO3936 wheat line, but not in the WT parent after re-watering of plants after 14 days water deficit treatment. Stomatal conductivity followed similar trends as that measured with chlorophyll fluorescence (**Figure 4B**).

**Figure 5:**
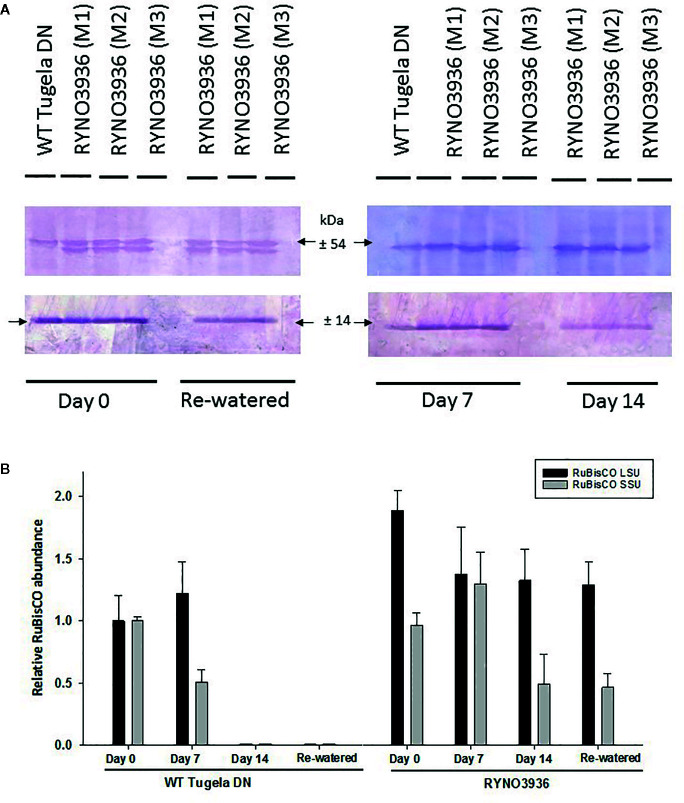
**(A)** Top: Protein blot of crude extract from WT Tugela DN and Mutant RYNO3936 wheat prior to (day 0) and after exposure (days 7 and 14) to water stress, and re-watered (day 21) probed with anti-Rubisco (LSU) and (SSU) IgG. All lanes were loaded with 20 mg total protein. Blots were probed with a 1:7 000 dilution of the polyclonal IgG against LSU, SSU. The leaf proteins were resolved by 12% (w/v) sodium dodecyl sulfate–polyacrylamide gel electrophoresis prior to transferring to nitrocellulose. Images were cropped for presentation purposes. M1–M3 represents three independent mutant lines. **(B)** Gel densitometric analysis of the protein blot in **(A)** of the rubisco large (LSU, 54 kDa) and small (SSU, 14 kDa) subunits in the leaf crude protein extracts from WT Tugela DN and Mutant RYNO3936 wheat prior to (day 0) and after exposure (days 7 and 14) to water stress, and re-watered. Data are expressed as relative levels of rubisco protein compared with the basic level in control line (mean value of 1.0). Each bar is the mean of three independent values (biological replicates) ± SE.

To confirm the observed changes in chlorophyll content and fluorescence in the WT Tugela DN and mutant RYNO3936 lines, the expression of RuBisCO before (day 0) and after induction of water deficit stress (days 7 and 14), and re-watering (day 21) was also analyzed using Western Blot analyses (**Figure 5**). Protein blots probed with anti-LSU (RuBisCO large subunit) and anti-SSU (RuBisCO small subunit) IgGs revealed two cross-reacting peptides for the large subunit (LSU) with sizes of 56 ± 4 kDa and 50 ± 4 kDa (LSU), respectively, and 15 ± 2 kDa for the small subunit, which corresponds to the sizes for the subunits in wheat (Botha and Small, 1987; Le Roux et al., 2019). Interestingly, a smaller form of the LSUs (50 ± 4 kDa) was observed in RYNO3936, but not in the WT, and this form disappears with the induction of water stress (days 7 and 14), but reappears after recovery (day 21, **Figure 5A**).

To further estimate the relative abundance of the RuBisCO subunits, the protein blots were scanned with a laser densitometer. Densitometric analyses of the blots revealed a high abundance in LSU in the mutant during irrigation, water stress, and rehydration, when compared to the WT (**Figure 5B**). Both subunits decreased in abundance with the induction of water deficit stress.

### Changes in Free Amino Acid Under Water Stress

To access the effect of water deficit on total free amino acid (FAA) the changes thereof were quantified in leaf material before (day 0) and after induction of water stress (days 7 and 14), and after re-watering (day 21; **Table 2**). Before the induction of water stress, the FAA levels between the WT Tugela DN and mutant lines were comparable, with RYNO3936 maintaining slightly lower FAA levels, except for methionine, leucine, and phenylalanine that were present in much higher levels in RYNO3936 (p < 0.05). Water deficit (day 7) induced higher amounts of proline, methionine, and phenylalanine in the WT Tugela DN, and higher amounts of serine, aspartate, glutamate, proline, lysine, and isoleucine in the mutant wheat plants. Prolonged water stress (day 14) further increased in all FAA (**Table 2**). Proline was the highest accumulated metabolite, during the water deficit conditions and tends to remain so despite re-watering conditions.

**Table 2:** Levels of free amino acids in leaf material of WT Tugela DN and Mutant RYNO3936 measured prior to (day 0) and after induction of water stress (days 7 and 14).

Genotype	Days	Free amino acid content [Concentration in % (m/m) dry solid]															
		his	ser	arg	gly	asp	glu	thr	ala	pro	lys	tyr	met	val	lle	leu	phe
**WT Tugela DN**	**0**	0.10	0.33	0.32	0.31	0.55	0.59	0.24	0.39	0.29	0.39	0.21	0.54	0.31	0.18	0.42	0.34
	**7**	nd	0.20	nd	0.27	nd	0.56	0.31	0.42	0.88	0.50	0.14	2.54	0.14	0.33	0.59	1.65
**RYNO3936**	**0**	0.10	0.20	0.20	0.16	0.53	0.26	0.10	0.19	0.15	0.17	nd	1.80	0.33	0.09	1.21	1.63
	**7**	0.17	0.30	0.27	0.29	1.10	0.90	0.19	0.34	1.30	0.39	0.19	0.91	0.39	0.23	0.50	0.46
	**14**	0.37	0.74	0.75	0.76	1.48	2.55	0.69	0.97	2.87	0.82	0.44	0.28	0.97	0.55	1.15	0.85
	**Re-watered**	0.37	0.20	0.14	0.18	0.42	0.36	0.10	0.22	0.43	0.23	0.14	0.56	0.23	0.10	0.25	0.79

Measurements were only taken for the WT Tugela DN until day 7, as it suffered irreversible damage and was dead by day 7 (n = 3). nd, not determined.

### Changes in the Proteome With Water Stress

**Figure 6:**
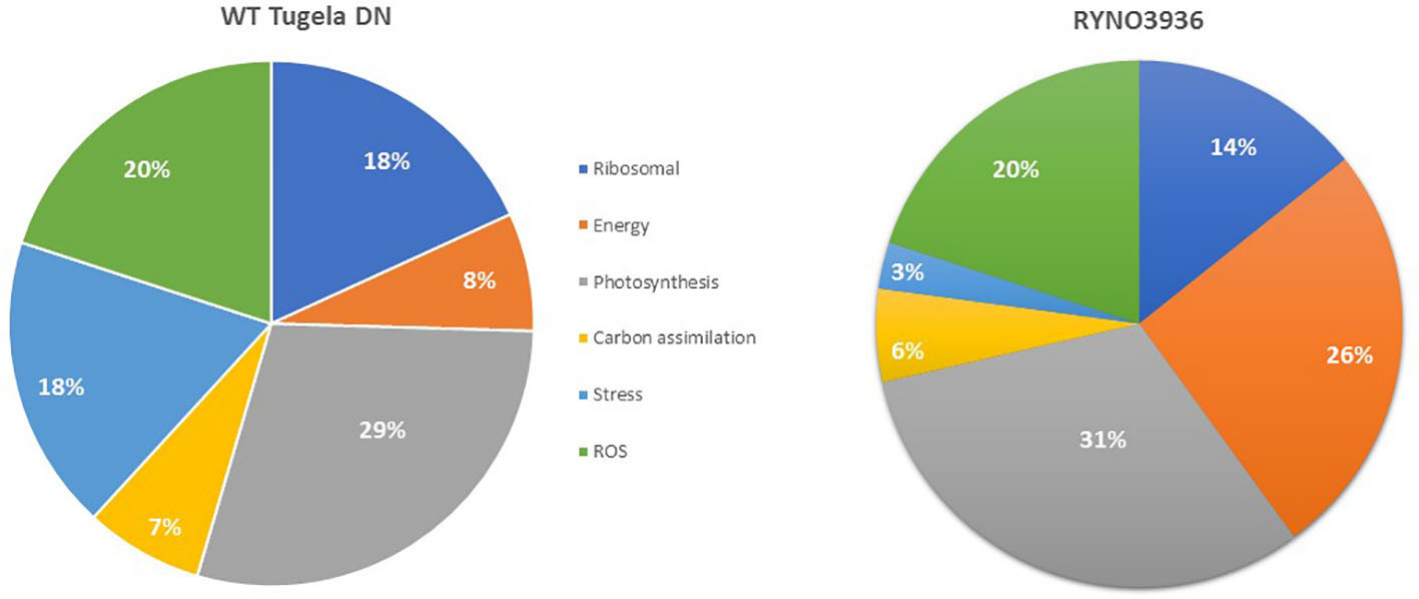
Comparison of the proteins expressed in WT Tugela DN (day 0) and the mutant RYNO3936 (day 0) according by the proportional contributions of functional categories.

In order to elucidate the changes in protein expression that was induced in RYNO3936 due to mutagenesis, we next compared the proteome of WT Tugela DN (day 0) with that of RYNO3936 (day 0) (**Figure 6**; **Supplementary Tables S1A, B**). When comparing the top 100 most significantly expressed proteins in WT Tugela DN with that in the mutant RYNO3936, most of the expressed proteins were expressed equally in both lines. However, there were unique proteins only expressed in RYNO3936 that have been previously identified in dehydration-tolerant plants (e.g., abscisic stress-ripening protein, cold induced protein, cold-responsive protein, dehydrin, and Group 3 late embryogenesis abundant protein) (**Supplementary Table S1**). Expressed proteins were grouped into key functional processes (i.e., ribosomal – protein synthesis; energy production – specifically ATP production; photosynthesis; carbon assimilation – specifically respiration; stress – includes host defense; and reactive oxidative stress (ROS) associated) to visualize the mutagenesis-induced differences that enable RYNO3936 to be more tolerant to water deficit than WT Tugela DN (**Figure 6**). RYNO3936 seemingly invests more resources into photosynthesis (e.g., chlorophyll a-b binding protein, Photosystem II CP47 chlorophyll apoprotein, Photosystem I P700 chlorophyll a apoprotein A2, Ribulose bisphosphate carboxylase large chain, Cytochrome b6-f complex iron-sulfur subunit, chloroplastic) and energy production (e.g., ATP synthase subunit alpha, mitochondrial) and less into the production of defense and stress-responsive proteins (e.g., lipoxygenase; glucanases) (**Table 3**).

**Table 3:** List of proteins expressed in RYNO3936 before (day 0) and after exposure to water stress (days 7 and 14), and recovery after re-watering (day 21) (*p* ≤ 0.05).

Identified Proteins (229)	Accession Number	Molecular Weight	ANOVA Test (p-value): *(p < 0.01463)
Oxygen-evolving enhancer protein 1, chloroplastic OS=Triticum urartu GN=TRIUR3_31979 PE=4 SV=1	M8AE10_TRIUA (+1)	34 kDa	<0.00010
ATP synthase delta chain, chloroplastic OS=Aegilops tauschii GN=F775_06392 PE=3 SV=1	N1R5T6_AEGTA	18 kDa	<0.00010
20 kDa chaperonin, chloroplastic OS=Aegilops tauschii GN=F775_32594 PE=3 SV=1	M8AVR4_AEGTA (+2)	26 kDa	<0.00010
Uncharacterized protein OS=Triticum aestivum PE=4 SV=1	W5GFX3_WHEAT (+1)	23 kDa	<0.00010
Stromal 70 kDa heat shock-related protein, chloroplastic OS=Aegilops tauschii GN=F775_29881 PE=3 SV=1	N1QXI7_AEGTA	73 kDa	<0.00010
Adenosylhomocysteinase OS=Triticum urartu GN=TRIUR3_03943 PE=3 SV=1	M7ZAK2_TRIUA (+2)	46 kDa	<0.00010
Alpha-amylase/trypsin inhibitor CM3 OS=Triticum aestivum PE=1 SV=1	IAAC3_WHEAT	18 kDa	<0.00010
Uncharacterized protein OS=Aegilops tauschii GN=F775_27291 PE=4 SV=1	M8CU50_AEGTA	32 kDa	<0.00010
UTP–glucose-1-phosphate uridylyltransferase OS=Triticum urartu GN=TRIUR3_15167 PE=4 SV=1	M7YXN3_TRIUA	51 kDa	<0.00010
40S ribosomal protein S20 OS=Triticum urartu GN=TRIUR3_10578 PE=3 SV=1	M7YRN1_TRIUA (+3)	14 kDa	<0.00010
Gamma-gliadin OS=Triticum dicoccoides GN=ll703 PE=4 SV=1	B6UKJ9_TRIDC (+13)	29 kDa	<0.00010
Aldehyde dehydrogenase 7b OS=Triticum aestivum PE=2 SV=1	D9IFB7_WHEAT (+1)	54 kDa	<0.00010
Uncharacterized protein OS=Triticum aestivum GN=TRAES_3BF028000060CFD_c1 PE=3 SV=1	A0A077S298_WHEAT (+1)	18 kDa	0.00015
Uncharacterized protein OS=Triticum aestivum PE=4 SV=1	W5DYH0_WHEAT	39 kDa	0.0002
Grain softness protein (Fragment) OS=Triticum aestivum GN=Gsp-1D PE=4 SV=1	A0A0A7AA82_WHEAT (+6)	16 kDa	0.00021
Lipoxygenase OS=Triticum aestivum PE=3 SV=1	W5F9D7_WHEAT	97 kDa	0.00023
5-methyltetrahydropteroyltriglutamate–homocysteine methyltransferase OS=Triticum urartu GN=TRIUR3_07672 PE=3 SV=1	M7ZHT1_TRIUA	85 kDa	0.00025
Fructose-bisphosphate aldolase OS=Triticum urartu GN=TRIUR3_24109 PE=3 SV=1	M7ZGS6_TRIUA (+3)	39 kDa	0.00032
ADP,ATP carrier protein, mitochondrial OS=Triticum urartu GN=TRIUR3_11472 PE=3 SV=1	M8A2G0_TRIUA (+3)	41 kDa	0.00032
Uncharacterized protein OS=Triticum aestivum PE=4 SV=1	W5FDV7_WHEAT	25 kDa	0.00037
Uncharacterized protein OS=Triticum aestivum PE=4 SV=1	W5HAX6_WHEAT	15 kDa	0.00037
Uncharacterized protein OS=Triticum aestivum PE=3 SV=1	W5FRF1_WHEAT	34 kDa	0.0004
Uncharacterized protein OS=Triticum aestivum PE=4 SV=1	W5B9F3_WHEAT	27 kDa	0.00054
Uncharacterized protein OS=Triticum aestivum PE=4 SV=1	W5G736_WHEAT	32 kDa	0.00054
Uncharacterized protein OS=Triticum aestivum PE=4 SV=1	W5HCY0_WHEAT	26 kDa	0.00069
Chlorophyll a-b binding protein, chloroplastic OS=Triticum urartu GN=TRIUR3_18701 PE=3 SV=1	M7YH23_TRIUA (+2)	38 kDa	0.00072
GTP-binding nuclear protein OS=Triticum urartu GN=TRIUR3_25734 PE=3 SV=1	M8ACU1_TRIUA (+5)	26 kDa	0.00076
60S acidic ribosomal protein P0 OS=Aegilops tauschii GN=F775_28558 PE=3 SV=1	N1QYE3_AEGTA	35 kDa	0.00086
Oxygen-evolving enhancer protein 3-1, chloroplastic OS=Aegilops tauschii GN=F775_30429 PE=4 SV=1	M8BB25_AEGTA (+3)	18 kDa	0.0013
Actin OS=Triticum aestivum PE=3 SV=1	A0A067YNJ5_WHEAT (+16)	42 kDa	0.0013
Low molecular weight glutenin OS=Triticum aestivum GN=Glu-A3 PE=4 SV=1	C3VN75_WHEAT (+3)	35 kDa	0.0013
Uncharacterized protein OS=Triticum aestivum GN=TRAES_3BF167600010CFD_c1 PE=4 SV=1	W5D1Z1_WHEAT	21 kDa	0.0014
30S ribosomal protein 1, chloroplastic OS=Aegilops tauschii GN=F775_31789 PE=4 SV=1	M8CEC3_AEGTA (+3)	26 kDa	0.0014
Photosystem II CP47 chlorophyll apoprotein OS=Aegilops tauschii GN=F775_04233 PE=4 SV=1	M8CB07_AEGTA	55 kDa	0.0015
70 kDa heat shock protein OS=Triticum aestivum PE=2 SV=1	C7ENF7_WHEAT (+1)	74 kDa	0.0015
Peptidyl-prolyl cis-trans isomerase OS=Triticum aestivum PE=2 SV=1	A7LM55_WHEAT	18 kDa	0.0016
Quinone oxidoreductase-like protein OS=Aegilops tauschii GN=F775_07275 PE=4 SV=1	M8AW52_AEGTA	32 kDa	0.002
Uncharacterized protein OS=Triticum aestivum PE=4 SV=1	W5HLU5_WHEAT (+1)	48 kDa	0.0021
Superoxide dismutase [Cu-Zn] OS=Triticum aestivum GN=SOD1.2 PE=2 SV=1	O24400_WHEAT	20 kDa	0.0022
Single-stranded nucleic acid binding protein OS=Triticum aestivum GN=whGRP-1 PE=2 SV=1	Q41518_WHEAT	16 kDa	0.0022
Low molecular weight glutenin subunit (Fragment) OS=Thinopyrum ponticum x Triticum aestivum PE=4 SV=1	Q5PU42_9POAL	34 kDa	0.0022
Uncharacterized protein OS=Triticum aestivum PE=3 SV=1	W5EHT8_WHEAT	19 kDa	0.0023
Photosystem I P700 chlorophyll a apoprotein A2 OS=Triticum aestivum GN=psaB PE=3 SV=1	PSAB_WHEAT (+1)	83 kDa	0.0026
Uncharacterized protein OS=Triticum urartu GN=TRIUR3_33029 PE=4 SV=1	M7Z7B4_TRIUA	32 kDa	0.0032
Uncharacterized protein OS=Aegilops tauschii GN=F775_04480 PE=4 SV=1	M8BDP7_AEGTA	26 kDa	0.0034
Uncharacterized protein OS=Triticum aestivum PE=3 SV=1	W5E575_WHEAT	35 kDa	0.0034
60S ribosomal protein L4-1 OS=Triticum urartu GN=TRIUR3_35018 PE=4 SV=1	M8A580_TRIUA (+2)	36 kDa	0.0037
Ribulose bisphosphate carboxylase large chain OS=Triticum aestivum GN=rbcL PE=1 SV=2	RBL_WHEAT (+1)	53 kDa	0.0045
Ribosomal protein OS=Triticum urartu GN=TRIUR3_07435 PE=3 SV=1	M7ZM70_TRIUA	18 kDa	0.0047
Cold-responsive protein WCOR14 OS=Aegilops tauschii GN=F775_26151 PE=4 SV=1	C0L981_AEGTA (+2)	14 kDa	0.0049
Putative calcium-binding protein CML7 OS=Triticum urartu GN=TRIUR3_30313 PE=3 SV=1	M7ZNI1_TRIUA	32 kDa	0.0057
Germin-like protein 8-14 OS=Triticum urartu GN=TRIUR3_27105 PE=3 SV=1	M7ZSU0_TRIUA (+2)	22 kDa	0.006
Glyceraldehyde-3-phosphate dehydrogenase OS=Aegilops tauschii GN=F775_07657 PE=3 SV=1	M8C8G6_AEGTA (+1)	37 kDa	0.0061
Uncharacterized protein OS=Triticum aestivum PE=4 SV=1	W5FGX7_WHEAT	41 kDa	0.0076
ATP synthase subunit alpha, mitochondrial OS=Triticum aestivum GN=ATPA PE=3 SV=1	ATPAM_WHEAT (+1)	55 kDa	0.0078
Uncharacterized protein OS=Triticum aestivum PE=3 SV=1	W5G312_WHEAT	82 kDa	0.008
Thioredoxin OS=Triticum urartu GN=TRIUR3_30421 PE=3 SV=1	M8ASF0_TRIUA (+5)	12 kDa	0.008
Uncharacterized protein OS=Triticum aestivum GN=TRAES_3BF092100100CFD_c1 PE=3 SV=1	A0A077S2R7_WHEAT	111 kDa	0.0087
Dimeric alpha-amylase inhibitor OS=Triticum aestivum PE=4 SV=1	I6PZ03_WHEAT (+1)	15 kDa	0.01
Uncharacterized protein OS=Triticum aestivum PE=3 SV=1	W5BD57_WHEAT	102 kDa	0.013
Cytochrome b6-f complex iron-sulfur subunit, chloroplastic OS=Triticum aestivum GN=petC PE=2 SV=1	UCRIA_WHEAT	24 kDa	0.013
Chloroplast inositol phosphatase-like protein OS=Triticum aestivum PE=2 SV=1	Q5XUV3_WHEAT (+1)	32 kDa	0.013
Putative vacuolar defense protein OS=Triticum aestivum GN=PR4e PE=4 SV=1	Q6PWL8_WHEAT (+1)	18 kDa	0.013
14-3-3 protein OS=Triticum aestivum GN=14R2 PE=2 SV=1	L0GED8_WHEAT (+3)	29 kDa	0.014
Globulin 1 OS=Triticum aestivum PE=4 SV=1	Q0Q5D4_WHEAT (+1)	25 kDa	0.014
Uncharacterized protein OS=Aegilops tauschii GN=F775_28677 PE=4 SV=1	R7W586_AEGTA	24 kDa	0.014
Chlorophyll a-b binding protein, chloroplastic OS=Triticum aestivum GN=CBP5 PE=2 SV=1	C1K5B9_WHEAT (+2)	29 kDa	0.015
Uncharacterized protein OS=Triticum aestivum GN=TRAES_3BF068000010CFD_c1 PE=4 SV=1	A0A077RQ49_WHEAT	274 kDa	0.015
ATP-dependent zinc metalloprotease FTSH 1, chloroplastic OS=Triticum urartu GN=TRIUR3_31373 PE=3 SV=1	M8ADT2_TRIUA (+2)	54 kDa	0.015
30S ribosomal protein 2, chloroplastic OS=Aegilops tauschii GN=F775_28246 PE=4 SV=1	M8BN30_AEGTA (+1)	20 kDa	0.015
Fructose-bisphosphate aldolase OS=Triticum aestivum GN=AlDP PE=2 SV=1	C0KTA6_WHEAT (+1)	42 kDa	0.016
Elongation factor Tu OS=Triticum urartu GN=TRIUR3_34609 PE=3 SV=1	M7ZEC4_TRIUA (+2)	46 kDa	0.017
Uncharacterized protein OS=Triticum urartu GN=TRIUR3_27117 PE=3 SV=1	M8AJF1_TRIUA (+3)	26 kDa	0.017
Glyceraldehyde-3-phosphate dehydrogenase OS=Aegilops tauschii GN=F775_05242 PE=3 SV=1	M8C9Y7_AEGTA (+2)	43 kDa	0.018
ATP synthase subunit alpha OS=Triticum urartu GN=atpA PE=3 SV=1	M8AUX6_TRIUA	62 kDa	0.019
Plastocyanin OS=Triticum aestivum PE=3 SV=1	W5DL22_WHEAT	16 kDa	0.02
Peroxisomal (S)-2-hydroxy-acid oxidase GLO1 OS=Triticum urartu GN=TRIUR3_22574 PE=4 SV=1	M7YXL1_TRIUA (+2)	40 kDa	0.021
Ribulose bisphosphate carboxylase small chain OS=Triticum urartu GN=TRIUR3_12281 PE=3 SV=1	M7YCR2_TRIUA (+7)	19 kDa	0.023
Glycine cleavage system H protein, mitochondrial OS=Triticum urartu GN=TRIUR3_12946 PE=3 SV=1	M7Z6F5_TRIUA	17 kDa	0.023
ATP synthase subunit OS=Triticum aestivum PE=2 SV=1	D3K4D8_WHEAT (+1)	40 kDa	0.023
Photosystem II CP43 reaction center protein (Fragment) OS=Triticum timopheevii GN=psbC PE=3 SV=1	A0A090ARF9_TRITI (+3)	54 kDa	0.024
Uncharacterized protein OS=Triticum aestivum PE=4 SV=1	W4ZSM1_WHEAT	15 kDa	0.025
Chlorophyll a-b binding protein, chloroplastic OS=Triticum urartu GN=TRIUR3_20986 PE=3 SV=1	M7ZM86_TRIUA (+3)	23 kDa	0.026
Uncharacterized protein OS=Triticum aestivum PE=4 SV=1	W5F6W2_WHEAT	16 kDa	0.027
LEA3 OS=Triticum turgidum subsp. durum PE=2 SV=1	A0A0M4HM24_TRITD	22 kDa	0.027
Uncharacterized protein OS=Aegilops tauschii GN=F775_29168 PE=4 SV=1	M8BNU5_AEGTA	91 kDa	0.028
Alanine aminotransferase 2 OS=Aegilops tauschii GN=F775_30987 PE=4 SV=1	M8BWQ5_AEGTA (+2)	58 kDa	0.028
Serine hydroxymethyltransferase OS=Triticum aestivum PE=3 SV=1	W5ECJ8_WHEAT	59 kDa	0.029
60S ribosomal protein L23a OS=Triticum urartu GN=TRIUR3_20390 PE=3 SV=1	M8A553_TRIUA (+2)	20 kDa	0.029
Cytochrome f OS=Triticum aestivum GN=petA PE=3 SV=3	CYF_WHEAT (+3)	35 kDa	0.03
High molecular weight glutenin subunit Ax-dp (Fragment) OS=Triticum polonicum PE=4 SV=1	D1MJA1_9POAL	91 kDa	0.03
Ribulose bisphosphate carboxylase small chain OS=Triticum aestivum GN=rbcS PE=3 SV=1	Q9FEE4_WHEAT	19 kDa	0.035
High molecular weight glutenin subunit OS=Triticum aestivum GN=Glu PE=4 SV=1	W6AWK6_WHEAT	67 kDa	0.035
Photosystem 1 subunit 5 OS=Triticum aestivum GN=pssv-1B PE=4 SV=1	Q2L3V4_WHEAT	15 kDa	0.036
Histone H2A OS=Aegilops tauschii GN=F775_29407 PE=3 SV=1	R7WEH7_AEGTA (+1)	16 kDa	0.039
Uncharacterized protein OS=Triticum aestivum PE=3 SV=1	W5B4C8_WHEAT (+1)	29 kDa	0.04
Fructose-bisphosphate aldolase OS=Triticum aestivum PE=3 SV=1	W5G4A2_WHEAT	42 kDa	0.041
Uncharacterized protein OS=Triticum urartu GN=TRIUR3_14987 PE=4 SV=1	M7ZIY8_TRIUA (+1)	27 kDa	0.044
RUBISCO activase alpha (Fragment) OS=Triticum aestivum GN=rca2_alpha PE=4 SV=1	A0A078BQY4_WHEAT (+3)	45 kDa	0.045
Cell division protease ftsH-like protein, chloroplastic OS=Aegilops tauschii GN=F775_28819 PE=3 SV=1	M8BVC8_AEGTA	72 kDa	0.048
PsbP-like protein 1, chloroplastic OS=Aegilops tauschii GN=F775_30938 PE=4 SV=1	R7VZH9_AEGTA (+2)	23 kDa	0.94

Indicated are the significantly regulated peptides, the accession number, molecular weight of the proteins, and P-values.

**Figure 7:**
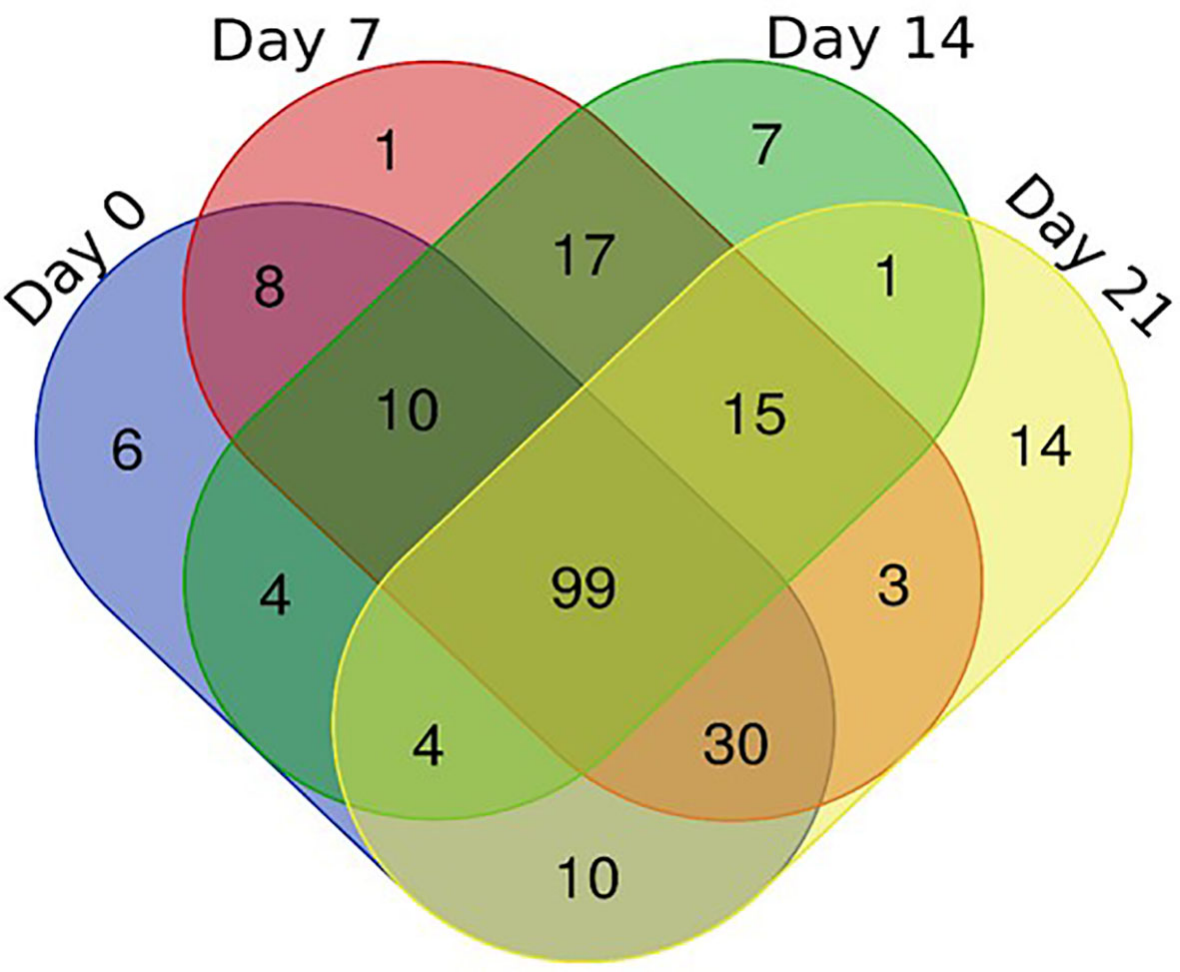
Venn diagram of the shared and unique proteins present in RYNO3936 before (day 0) and after exposure to water stress (days 7 and 14), and recovery after re-watering (day 21).

**Figure 8:**
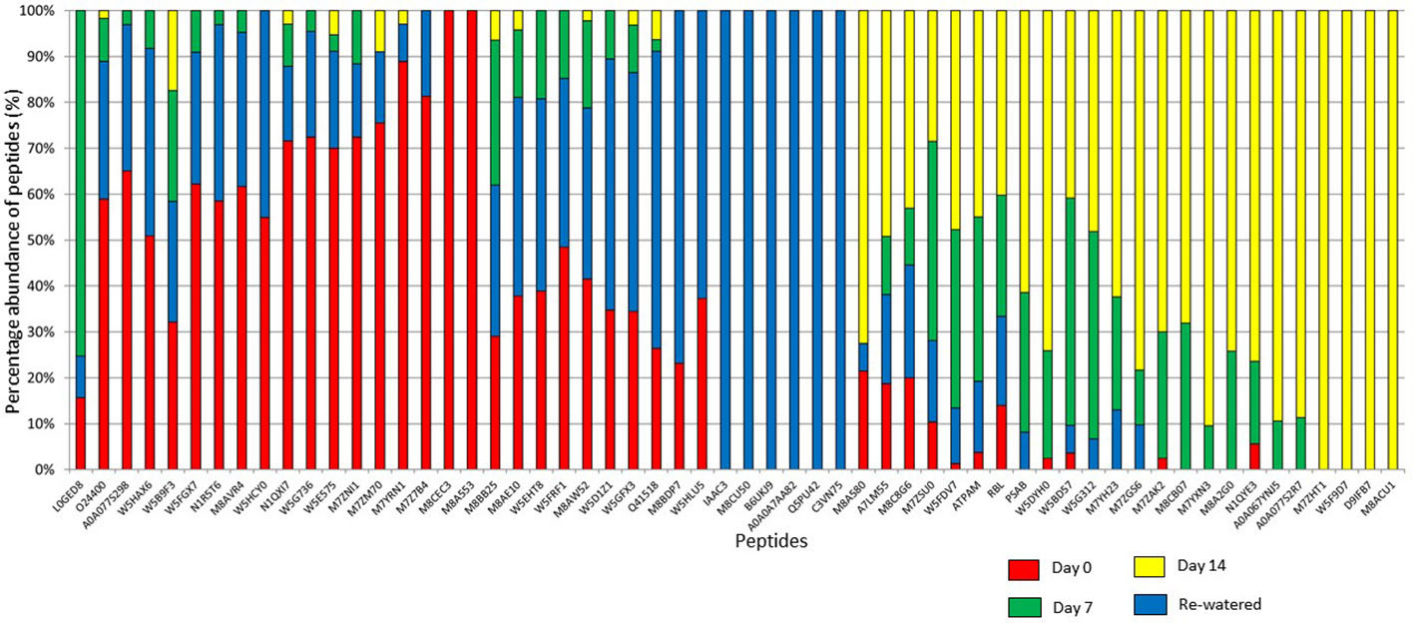
Percentage abundance of the Top 100 peptides expressed in RYNO3936 before (day 0) and after exposure to water stress (days 7 and 14), and recovery after re-watering (day 21).

To better understand the coping mechanisms applied by mutant RYNO3936, we also compared the changes in the proteome of RYNO3936 that occurred before (day 0) and after induction of water stress (days 7, 14), and recovery of the mutant plant (day 21; **Supplementary Tables S1**, **S2**, **S3**). After analysis of the proteins in RYNO3936 across all treatments, we found that 99 proteins were shared among all treatments (**Figure 7**, **Supplementary Table 1S**), with 6 uniquely expressed at day 0 (e.g., thioredoxin, cold responsive proteins), only 1 on day 7 (i.e., LAlv9 family protein), a total of 7 on day 14 (e.g., aldehyde dehydrogenase, lipoxygenase, GTP-binding nuclear protein, heat shock protein 81, 5-methyltetrahydropteroyltriglutamate-homocysteine methyltransferase), and 14 proteins in re-watered, recovered leaf material (day 21) (e.g., alpha gliadin, alpha-amylase/trypsin inhibitor, globulin, gamma gliadin, grain softness protein, high and low molecular weight glutenin subunit proteins, LEA3 protein, dimeric alpha-amylase inhibitor, grain softness protein) (**Figure 8**).

To visualize the observed proteomic changes before (day 0), after induction of water stress (days 7 and 14) and after recovery (day 21), we conducted a cluster analysis (Eisen et al., 1998) and visualized the clusters using TreeView (Saldanha, 2004) (**Supplementary Figure S1**, **Supplementary Table S2**). We obtained two clusters with two major groupings according to protein expression patterns, with unstressed (day 0) and recovered plants (day 21) that grouped together, and stressed plants (days 7 and 14) that formed another grouping.

We also assigned the obtained peptides into functional categories (**Supplementary Figure S2**) to confirm their involvement in plant metabolism. Within the biological processes, most of the peptides belonged to cellular metabolic process (18%), while in the cellular component the largest groups belonged to the intracellular (25%) and intercellular (25%) parts. When assigned to the molecular function, most peptides belonged to the ion binding category (38%).

### Protein Turnover During Water Deficit

**Figure 9:**
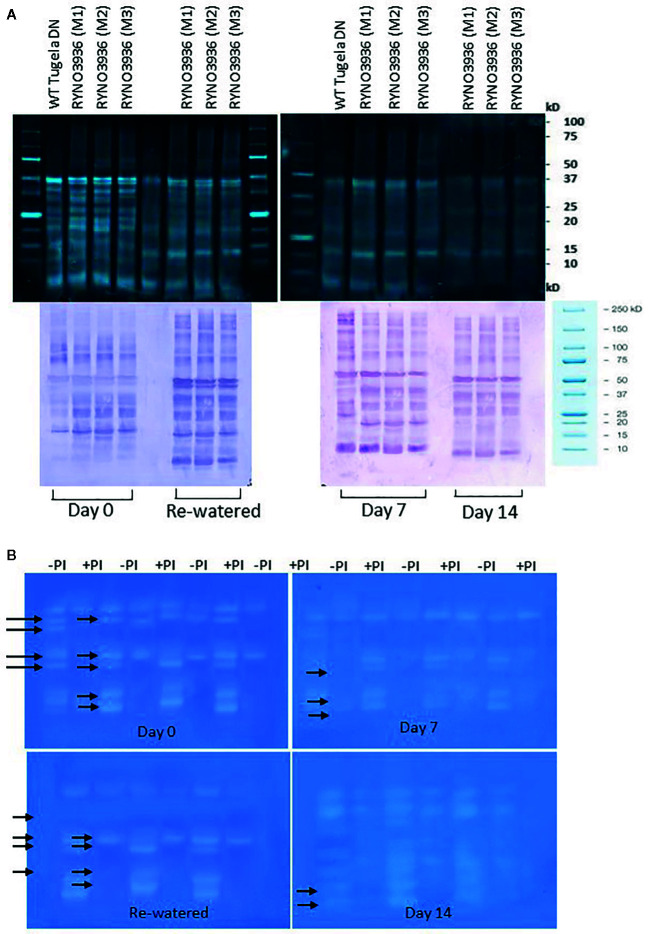
**(A)** Top: Crude protein separated on a 12.5% SDS-PAGE from WT Tugela DN and Mutant RYNO3936 wheat prior to (day 0) and after exposure to water stress (days 7 and 14). All lanes were loaded with 20 μg total protein. Bottom: WT Tugela DN and Mutant RYNO 3936 wheat prior to (day 0) and after exposure (days 7 and 14) to water stress, probed with anti-SUMO IgG. All lanes were loaded with 20 μg total protein. Blots were probed with a dilution of 1:10 000 dilution of monoclonal IgG against SUMO1. Images were cropped for presentation purposes. M1 to M3 represents three independent Mutants. **(B)** Gradient Zymograms depicting proteolytic activity of WT Tugela DN and Mutant RYNO3936 prior to (day 0) and after exposure to water stress (days 7 and 14). Zymograms (gradient, 5–15%) were cast and in all cases 35 μg protein was loaded. Inclusion of an incubation step with 0.1 mM Cysteine Protease inhibitor (E-64) performed at pH 7, enabled for the identification of cysteine proteases. Lanes with + PI refers to treatment with protease inhibitor; whereas - PI refers to no inhibitor treatment. Arrows indicated bands that were removed after treatment with the 0.1 mM Cysteine Protease inhibitor (E-64). The presented data is representative of two independent experiments. Images were cropped for presentation purposes, and the contrast was adjusted (10%).

To study the changes in SUMO cysteine proteases, blots of separated crude protein extracts were probed with monoclonal anti-SUMO1 IgG. Several cross-reacting peptides were found ranging in sizes from 150 ± 10 kDa to 10 ± 5 kDa. At day 0, both the WT Tugela DN and mutant RYNO3936 plants had one cross reacting SUMO1 peptide present that was absent in the other line (**Figure 9A**, bottom). At day 7, the WT Tugela DN had two peptides that were absent in the mutant RYNO3936, while the mutant plant had a cross-reacting SUMO1 peptide present that was absent in WT wheat line. When comparing the profile of cross-reacting SUMO1 peptides in RYNO3936 before (day 0) and after induction of water stress (days 7, 14), and after recovery (day 21), more differences in banding patters and intensity of the proteins were observed (**Figure 9A**, bottom).

To further elucidate whether the peptides on the protein blots were cysteine proteases, we included a protease inhibitor E64 specific to cysteine proteases during the protein analysis before separation on gradient zymograms (**Figure 9B**). The WT Tugela DN and mutant RYNO3936 plants differed in protein bands with proteolytic activity. Addition of the cysteine protease inhibitor E64 blocked the activity of three different proteases in each of the plants. A comparison between the profiles of the mutant plant before (day 0) and after induction of water stress (days 7, 14), and after recovery (day 21), revealed three protein bands with proteolytic activity in unstressed and recovered mutant plants, but four protein bands with proteolytic activity in the water stressed plants (days 7 and 14).

### Oxidative Defense Enzymes

**Figure 10:**
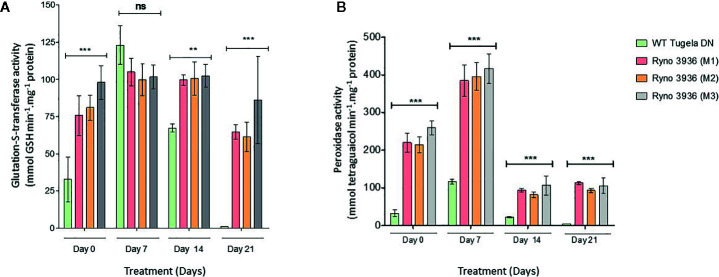
Changes in the peroxidase (POX) **(B)** and glutathione-S-transferase (GST) **(A)** activities measured in WT Tugela DN and Mutant RYNO 3936 wheat prior to (day 0) and after induction of water stress (days 7 and 14). POX activity was measured by the formation of tetraguaiacol monitored at 470 nm while GST activity represents the formation of GS-DNB conjugate at 340 nm. Capped bars above means represent ± SD of three replicates. Asterisks above columns means denote the significant differences compared with WT Tugela DN for a single mutant line. **P ≤ 0.01; ***P ≤ 0.05. ns, nonsignificant.

Mutant RYNO3936 expressed significantly higher POX activity than WT Tugela DN before (day 0) and after water stress (days 7 and 14), with the highest activity on day 7 after induction of water stress (**Figure 10A**). GST activity increased significantly in WT Tugela DN after induction of water deficit stress but did not change significantly in the mutant plant (P < 0.05) (**Figure 10B**).

## Discussion

Drought tolerance is a polygenic trait that is difficult to attain using conventional breeding. However, chemically induced mutagenesis has already produced excellent results by altering major polygenic traits leading to synergistic effects which increased the quality and yield of wheat (Ahloowalia et al., 2004; Tian et al., 2012). Inducing random mutagenesis in a red hard winter wheat line (Tugela DN), a mutant wheat line RYNO3936 with improved tolerance to water deficit, and the ability to recover fully and reproduce, after being physiologically “dead” (leaves completely dry/dehydrated) was developed. In the current study, we characterized the physiological and biochemical responses of this mutant wheat line RYNO3936 during irrigation and induced water deficit stress.

Our mutant's coping mechanism differs significantly from that of the WT wheat line, as prolonged exposure to water deficit stress seemingly has no influence on the phenotype of the mutant plant, while the WT wheat line suffered various developmental deformities (i.e., leaves are wrinkled or twisted and heads are deformed) and dies. Under non-water deficit conditions, the mutant plants took longer to reach maturity when compared to the control but produced more tillers and 4× more seed. Under water deficit stress, however, our mutant plants matured faster than WT Tugela DN and produced the same amount of seed than WT Tugela DN under irrigation. This seemingly rapid transition from a vegetative to reproductive stage corresponds to observation in engineered rice (Oryza sativa) expressing the galactinol synthase gene when exposed to water deficit conditions (Selvaraj et al., 2017), which essentially affords the plant the ability to produce seeds before the induced stress becomes lethal for the crop (Kazan and Lyons, 2016; Gol et al., 2017). Interestingly, the mutant RYNO 3936 avoids premature wilting during induced water stress, as demonstrated by its high RMC when compared to WT Tugela DN, which visually wilted within 3 days after water was withheld. Physiologically, the function of wilting is to soften leaves to enable leaf rolling, thus limiting surface area for further water loss by evaporation (Bowne et al., 2012). Mutant RYNO3936 expresses a “slow-wilting phenotype”, since it retains a higher RMC during water deficit stress and loses rigidity only with prolonged water stress (day 14). Even though RMC declines in the mutant and the plant shows visual senescence (browning of leaves), the visual browning is less when compared to that of WT Tugela DN. However, after re-watering, the mutant fully recovers as demonstrated by the increase in RMC, suggesting that the mutant can readjust its osmoregulation assuming full turgor pressure and recover to activate its metabolic activity providing whole-plant relief to the water deficit stress (Souza et al., 2004).

When water deficit stress was induced, both WT and mutant plants senesced with their leaves losing their green color which is synonymous to the preferential degradation of chlorophyll over carotenoids (Balazadeh, 2014; Li et al., 2019). The visible de-greening of the leaves, however, took longer in the mutant RYNO3936, as it was evident from its chlorophyll content that remained higher when compared to the WT Tugela DN. This observed “slow-wilting phenotype” is substantiated by the fact that the mutant remained photosynthetically active much longer than the WT Tugela DN (Thomas and Ougham, 2014), before it also senesces. A reduction of chlorophyll during water deficit stress is common in many crop species and largely dependent on the duration and severity of water stress (Brestic et al., 2015; Lakra et al., 2015; Peng et al., 2017).

It is evident that the mutant RYNO3936 could maintain an active metabolic state despite the experienced water deficit stress, as substantiated by the high rate of stomatal conductance and chlorophyll fluorescence during water deficit stress conditions. It is well understood that chlorophyll fluorescence and stomatal conductance are intrinsically coupled under a wide range of environmental conditions (i.e., soil moisture). However, different crop species such as tomatoes, kidney beans, potatoes, rice, and wheat manage the intrinsic relationship differently (Miyashita et al., 2005; Yuan et al., 2015; Ouyang et al., 2017; Wheeler et al., 2019; Wang et al., 2020). Generally, studies suggest that water deficit stress induces a reduction in photosynthesis and stomatal conductance, consequently, restricts the availability of CO2 for carboxylation (Ehonen et al., 2019). The mutant showed a decline in chlorophyll fluorescence and stomatal conductance only after prolonged induced water deficit stress (14 days) and restored to near full functionality with re-watering (Yi et al., 2016). The manner in which the mutant manifest its physiological response to water deficit and re-watering, is consistent with the results reported in maize inbred and hybrid lines (Chen et al., 2016) and two wheat cultivars (Abid et al., 2018), that showed a decrease in chlorophyll fluorescence and stomatal conductance during water deficit stress, but rapid “recovery” in chlorophyll fluorescence and stomatal conductance to normal levels after re-watering. This suggests that the stomatal aperture increased with re-watering thereby facilitating diffusion of CO2 from the atmosphere to the carboxylation site of RuBisCO (Galmés et al., 2011; Kumar et al., 2019).

Water deficit stress is usually associated with the onset of senescence. The process of senescence is associated with modification and/or degradation of photosynthetic proteins making them dysfunctional. Since random mutagenesis was applied to the mutants, it is likely that changes were induced in single/multiple genes, resulting in the expression of more proteins involved in photosynthesis and energy production. *Amaranthus hybridus* is an excellent example where a mutation in a chloroplast protein (i.e., a photosystem II electron transport protein that binds the electron carrier plastoquinone) improved tolerance to water deficit (Hirshberg and McIntosh, 1983). The plant also had higher photosynthetic rates when compared to control plants under water deficit conditions (Arntz et al., 2000).

To further our understanding of how the mutant plant manages its photosynthesis, we investigated RuBisCo protein expression. This enzyme is vital for CO_2_ fixation and oxygenation. The protein is assembled in the chloroplast from nuclear-encoded genes, cytosol synthesized small subunits (SSU) and plastid encoded genes which synthesis the large subunits (LSU) (Kawashima and Wildman, 1970; Ellis, 1979). Collectively the SSU is responsible for maintaining the form, structure, and activity of RuBisCo, whereas the LSU contain all the active sites (Knight et al., 1990; Andersson and Backlund, 2008). Not only does each subunit's response to water deficit stress differ, the response is also crop dependent. Mutant RYNO 3936 possesses a high abundance of LSU prior to water deficit stress, which only decreases upon the first 7 days of water stress thereafter a slight decline was observed (**Figures 9A, B**). Prolonged water deficit stress (day 14) followed by re-watering resulted in no significant changes in LSU abundance. This unique preservation of LSU could be due to effective control of proteases, which are able to degrade the large subunit of Rubisco (Bushnell et al., 1993). Prins et al. (2008) also found elevated levels of LSU in all tissue types of engineered tobacco (*Nicotiana tabacum* L.) that express a rice cystatin (a family of cysteine proteinases), when compared to WT tobacco. In contrast, the small subunit levels decreased after induction of water stress and never recovered to its pre-stressed state (**Figures 9A, B**) despite full recovery of the plant. Similar findings were also reported for tomato, Arabidopsis, and rice (Bartholomew et al., 1991; Williams et al., 1994; Vu et al., 1999).

Water stress induces senescence processes associated with protein degradation and turnover. We found changes in the mutant's protein profiles after induction of water stress (days 7, 14) and after recovery post-re-watering (day 21) which might also be associated with increased expression of SUMO-proteases with a papain-like proteinase fold (Hickey et al., 2012). In this regard, we found a protein of ± 50 kDa in size highly abundant in the mutant's profile after recovery post-re-watering (**Figure 9A**). Under non-stressed conditions SUMO tags may serve as priming sites essentially preparing our mutant for early stress recognition (Conrath et al., 2002). Many studies have also demonstrated that plants unable to latch SUMO1/2 onto substrate proteins show phenotypes with changes in flowering time (Castro et al., 2018), immune responses (Saleh et al., 2015), growth reduction, and reduced tolerance to salinity, drought, heat, freezing, and phosphate starvation (Roden et al., 2004; Catala et al., 2007; Miura et al., 2007; Li et al., 2019). Although SUMOylation is strongly associated with drought susceptibility (Benlloch and Lois, 2018), a mutated proteome, as in our case, may circumvent the deleterious SUMOylation effects by increasing SUMO-proteases for deSUMOylation influx. In a previous study, we concluded that overexpressing Overly Tolerant to Salt-1 in spring wheat (Gamtoos R) enhances water stress tolerance in the transgenic plants and significantly reduces the effects of SUMOylation (Le Roux et al., 2019). Since SUMO proteins belong to the family of cysteine protease (Botha et al., 2017), we investigated total proteases and cysteine proteases by using the inhibitor E64. Proteases play an important role in programmed cell death, senescence and protein remobilization, and have been shown to be induced by water stress (Liu et al., 2018). RYNO 3936 had fewer and a lower abundance of cysteine proteases during water stress. This also indicates that the mutant has less protein degradation under water deficit stress and therefore senesce less (Botha et al., 2017; Le Roux et al., 2019).

We further found in our study that our mutant plants differ significantly in antioxidative enzyme activities before (day 0) and after induction of water stress (days 7, 14) when compared to WT Tugela DN plants. Water stress generally leads to more ROS signaling linked to ABA production, Ca2+ fluxes and sugar sensing (Cruz de Carvalho, 2008 and references within). In our study, we specifically found expression of an abscisic stress-ripening protein in our mutant plant which was absent from the WT control indicating a greater control to manage increasing ROS production.

The mutant also expressed high levels of POX and GST, in particular 7 days after initiation of water deficit stress. Such increase in POX was also recently found in two drought-tolerant Chinese wheat varieties when exposed to water stress (Abid et al., 2018), as well as in the tasg1 wheat mutant (Tian et al., 2012). POX catalyzes hydrogen peroxide-dependent oxidation of a wide range of substrates, mainly phenol derivatives (Noctor et al., 2018; Smirnoff and Arnaud, 2019). GST provides a unique intracellular protection and an increase in GST is linked with sustaining cell redox homeostasis and guarding organisms against oxidative stress (Chen et al., 2012; Kumar and Trivedi, 2018). Overall, our findings suggest that there is a significant difference in antioxidative enzyme activities before (day 0) and after induction of water deficit stress (days 7, 14) when compared to WT Tugela DN plants. Water stress generally leads to more ROS signaling linked to ABA production, Ca2+ fluxes, and sugar sensing (Cruz de Carvalho, 2008 and references within). In our study, we specifically found expression of an abscisic stress-ripening protein in our mutant plant which was absent from the WT control indicating a greater control to manage increasing ROS production.

Increases in GST and POX activity further coincided in our study with a higher content of FAA with proline being the highest (**Table 2**), which might suggest that proline also participates in scavenging reactive oxygen species in addition to its role as an osmolyte as previously reported for salt-stressed plants (Hoque et al., 2007; Hossain et al., 2011; Cruz de Carvalho et al., 2013; Rejeb et al., 2014). Proline provides osmo-protection and the amount increases in many plant species, including maize, wheat, and pea, following exposure to water deficit stress (Matysik et al., 2002; Rampino et al., 2006; Charlton et al., 2008; Szabados and Savoure, 2010; Witt et al., 2012; Abid et al., 2018; Le Roux et al., 2019; Verslues and Juenger, 2019). Other FAAs that were much higher in the mutant when compared to the WT Tugela DN include methionine, asparagine, isoleucine, and phenylalanine. Pool sizes of FAAs are important, not only because of their requirement in protein biosynthesis (Good and Zaplachinski, 1994), but also for their additional functions in plant metabolism and signal transduction processes which ultimately may contribute to adequate water stress response. The extended level of amino acids is evident in enhancing stress pliability in our study, since RYNO3936 increases FAA under water deficit stress conditions, but FAA concentrations decrease considerably after re-watering to near well-watered levels (day 0). Abid et al. (2018) demonstrated that drought stress leads to a gradual increase of FAA, but soon decrease to match that of well-watered levels in wheat. This effective management of FAA has been suggested to aid in drought tolerance by protein stabilization, ROS detoxification and osmotic adjustment (Bowne et al., 2012; Rabara et al., 2017; Michaletti et al., 2018; Jia et al., 2019). FAA pool sizes are known to be induced under stress and during senescence (Hildebrandt et al., 2015 and references within). More important in the context of this study, in a recent study by Yadav et al. (2019), a strong genetic association was observed between glasshouse-based RWC, metabolites, and yield gap-based drought tolerance (YDT; the ratio of yield in water-limited versus well-watered conditions) across 18 field environments spanning sites and seasons. Specifically of interest is the observation that 98% of the genetic YDT variance could be explained by drought responses of four metabolites: serine, asparagine, methionine, and lysine (R2 = 0.98; P < 0.01). More specifically, that higher levels of methionine and lysine were more strongly associated with higher YDT, than the other amino acids, which support our observations in RYNO3638 with enhanced water deficit stress tolerance (Le Roux et al., 2019).

Changes in the transcriptome differs to changes at protein level (Gygi et al., 1999; Bogeat-Triboulot et al., 2006; Morimoto and Yahara, 2018), necessitating studies into the proteome to elucidate the water stress response pathway in crop species (Ford et al., 2011; Budak et al., 2013; Rabello et al., 2014; Liu et al., 2015; Chmielewska et al., 2016). In our study, we also found that our mutant RYNO3936 expressed proteins associated with osmotic stress tolerance (e.g. abscisic stress-ripening protein, cold induced protein, cold-responsive protein, dehydrin, and Group 3 late embryogenesis abundant protein, LEA) more than in the control. Dehydrin and LEA proteins have been long known to increase osmotic stress tolerance in plants (Figueras et al., 2005; Brini et al., 2007). With induction of water stress (day 7), the mutant expressed a LAlv9 family protein belonging to the family of lipocalins. Literature is rather limited regarding functionality of these proteins. In the past, lipocalins have been classified as transport proteins; however, it is now clear that lipocalins are involved in a variety of functions, including regulation of cell homeostasis, modulation of the immune response, programmed cell death (Lee et al., 2007; Kale et al., 2018) and, as carrier proteins, to act in the general clearance of endogenous and exogenous compounds (Flower, 1996 and references within; Kale et al., 2018). In mammals, an ortholog of the lipocalin family (BCL-2) controls cell death primarily by direct binding interactions that regulate mitochondrial outer membrane permeabilization (MOMP) leading to the irreversible release of intermembrane space proteins, subsequent caspase activation, and apoptosis (Kale et al., 2018). While in plants, the AtTIL lipocalin was shown to be functional in modulating tolerance to oxidative stress (Charron et al., 2008), when it was demonstrated that overexpression enhances tolerance to stress caused by freezing, paraquat, and light in Arabidopsis by encoding components of oxidative stress and energy balance. More importantly in the context of the present study, in Arabidopsis AtTIL lipocalin delays flowering and maintains leaf greenness in the latter plant, like our observations in RYNO3936. So lipocalin might partly explain the more water deficit stress tolerance of the mutant. This would be a new and important aspect not found so far for a wheat mutant with improved drought tolerance.

In conclusion, we characterized a new wheat mutant RYNO3936 which is associated with delayed water deficit stress-induced leaf senescence and rapid drought recovery. In particular, when this mutant was exposed to water deficit conditions it displayed higher RWC in its roots and leaves, sustained its chlorophyll fluorescence activity and stomatal conductance, accumulated in a selected set of FAAs associated with drought stress, expressed a unique set of proteins, showed delayed protein degradation and higher antioxidative enzyme activities when compared with its WT progenitor. Our mutant also uniquely expressed an abscisic stress-ripening protein and a LAlv9 family protein belonging to the family of lipocalins, which are involved in a variety of functions, including regulation of cell homeostasis, modulation of the immune response, and programmed cell death. Overall, our results suggest that our new high yielding wheat mutant RYNO2936 has a potential application in wheat breeding programs to enhance drought tolerance. Additionally, we characterized several of its unique traits (compared to the WT) that will assist future screening of mutant germplasm.

## Data Availability Statement

The mass spectrometry proteomics data have been deposited to the ProteomeXchange Consortium *via* the PRIDE (Perez-Riverol et al., 2019; http://www.ebi.ac.uk/pride) partner repository with the dataset identifier PXD019464 and 10.6019/PXD019464.

## Author Contributions

M-SR and A-MB-O planned the study. M-SR measured the plant phenotypes, analyzed chlorophyll fluorescence and stomatal conductance, as well as protein extractions for gel electrophoresis, and Western blots. A-MB-O conducted the enzyme assays. A-MB-O and M-SR extracted the proteins for MS/MS analysis, while MV conducted the MS/MS analysis. NB assisted with the bioinformatics and cluster analysis. M-SR and A-MB-O wrote the first draft. KK, MV, NB, and CC contributed to the interpretation of data and made editorial inputs.

## Conflict of Interest

The authors declare that the research was conducted in the absence of any commercial or financial relationships that could be construed as a potential conflict of interest.

## Supplementary Material

Table S2 Blast2GO results from the peptides obtained after LC-ESI-MS/MS analysis of total protein isolated from RYNO3936 before (day 0), and after induction of water stress (days 7 and 14), and after recovery and regrowth (day 21).

Table S3 List of proteins expressed in RYNO3936 before (day 0), and after induction of water stress (days 7 and 14), as well as after recovery and regrowth (day 21). Indicated are the sequence name, protein identity, length and number of hits, number of GO terms, and *e*-value. The cluster numbers correspond to that given in **Figure S2**.

Figure S1 Proportional contribution of proteins expressed in RYNO3936 to the different functional categories, where **(A)** biological processes; **(B)** cellular component; and **(C)** molecular function.

Figure S2 Cluster image generated by Java TreeView (Saldanha, 2004) of the proteins obtained after LC-ESI-MS/MS analysis of total protein isolated from RYNO3936 before (day 0), and after induction of water stress (days 7 and 14), and after recovery and regrowth (day 21). Red bands show up-regulated proteins, whereas green bands show down-regulated proteins.
