# Supplementary figures and images for: Wheat Line “RYNO3936” Is Associated With Delayed Water Stress-Induced Leaf Senescence and Rapid Water-Deficit Stress Recovery
Source: Front Plant Sci. 2020 Jul 14;11:1053. doi: 10.3389/fpls.2020.01053 (PMC7372113; doi:10.3389/fpls.2020.01053)

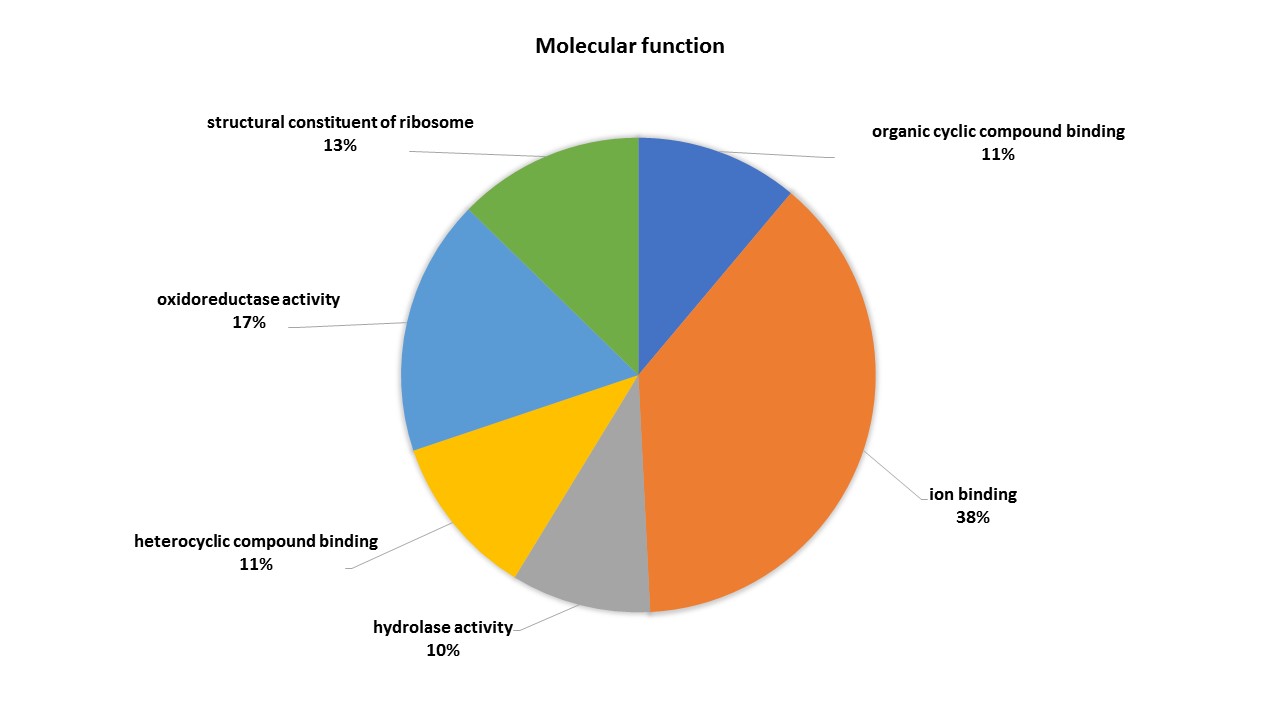

Supplement: Figure S1 — Proportional contribution of proteins expressed in RYNO3936 to the different functional categories, where (A) biological processes; (B) cellular component; and (C) molecular function. [file Image_1.jpeg]

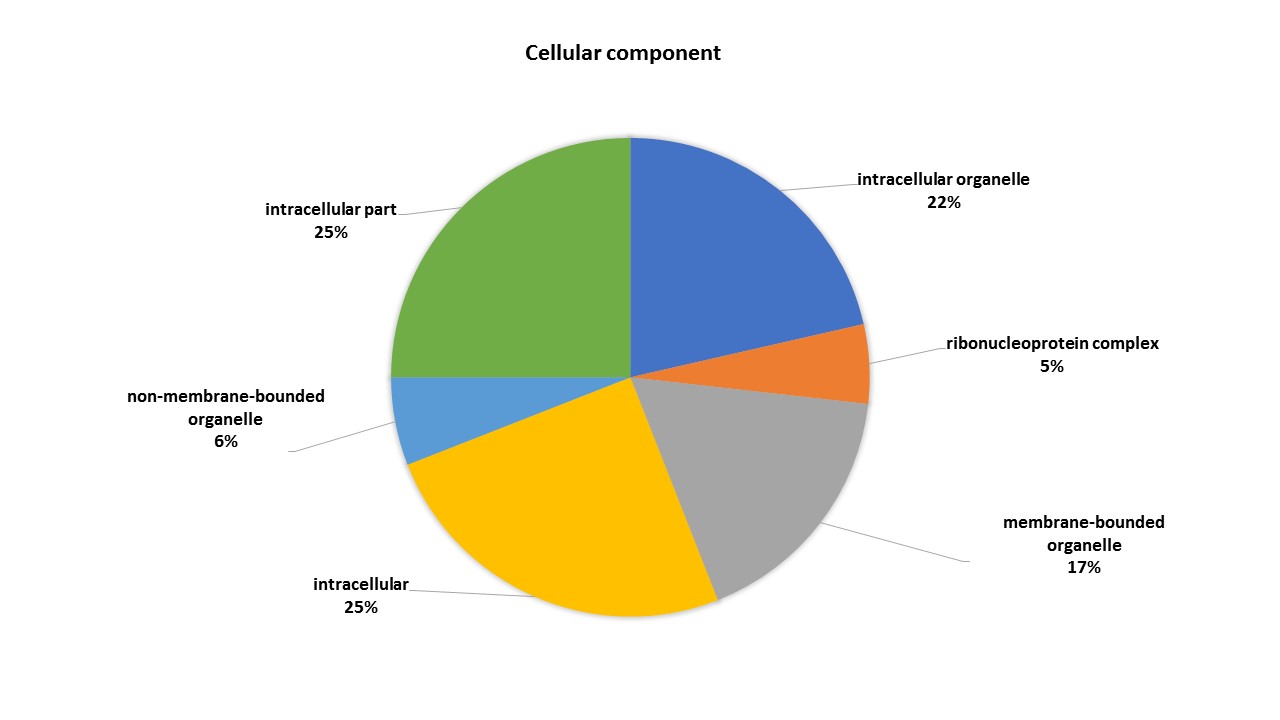

Supplement: Figure S2 — Cluster image generated by Java TreeView (Saldanha, 2004) of the proteins obtained after LC-ESI-MS/MS analysis of total protein isolated from RYNO3936 before (day 0), and after induction of water stress (days 7 and 14), and after recovery and regrowth (day 21). Red bands show up-regulated proteins, whereas green bands show down-regulated proteins. [file Image_2.jpeg]

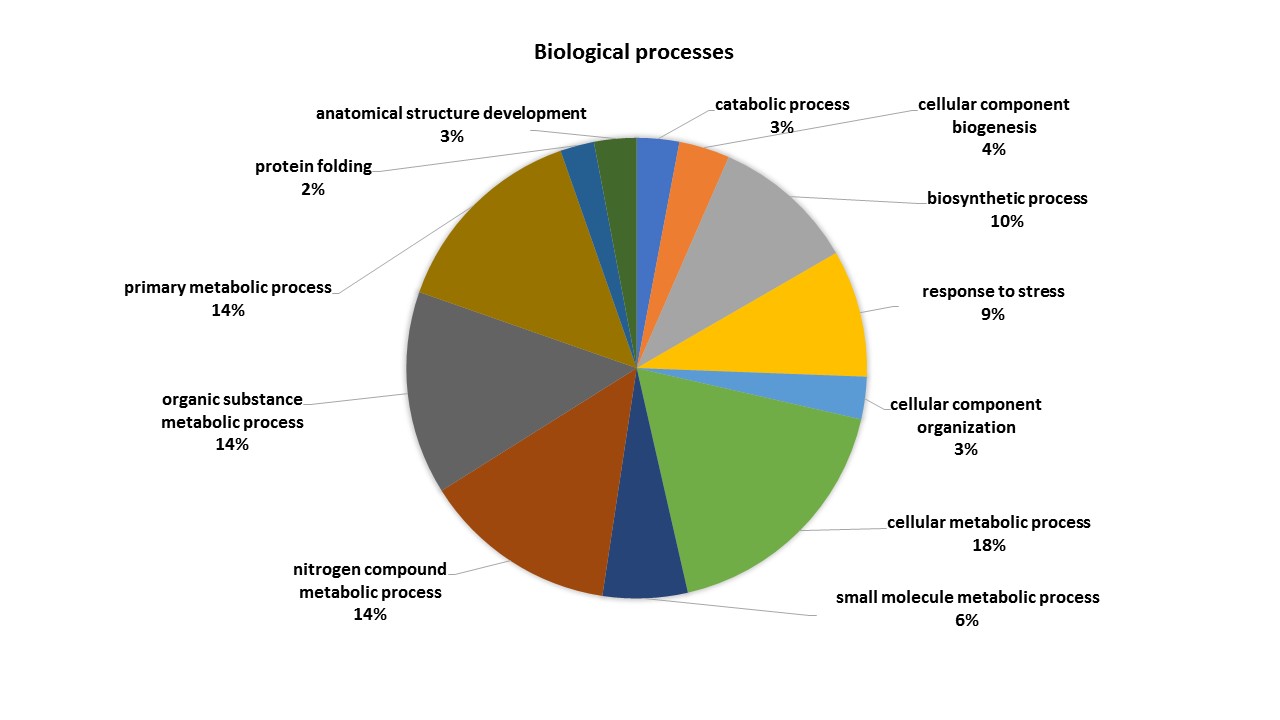

Supplement: Supplementary file 4 [file Image_3.jpeg]

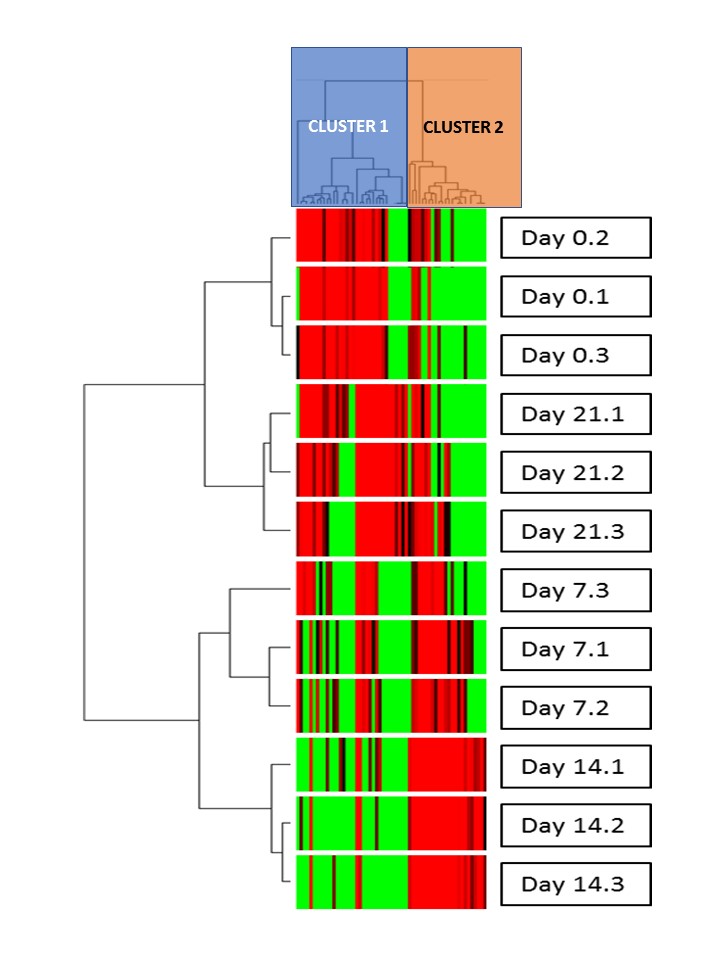

Supplement: Supplementary file 5 [file Image_4.jpeg]
